# Responses of soil microbiome to steel corrosion

**DOI:** 10.1038/s41522-020-00175-3

**Published:** 2021-01-21

**Authors:** Ye Huang, Dake Xu, Lu-yao Huang, Yun-tian Lou, Jiang-Baota Muhadesi, Hong-chang Qian, En-ze Zhou, Bao-jun Wang, Xiu-Tong Li, Zhen Jiang, Shuang-Jiang Liu, Da-wei Zhang, Cheng-Ying Jiang

**Affiliations:** 1 State Key Laboratory of Microbial Resources, Institute of Microbiology, Chinese Academy of Sciences, Beijing, 100101 China; 2 University of Chinese Academy of Sciences, Beijing, 100049 China; 3 Shenyang National Laboratory for Material Sciences, Northeastern University, Shenyang, 110819 China; 4 Beijing Advanced Innovation Center for Materials Genome Engineering, National Materials Corrosion and Protection Data Center, Institute for Advanced Materials and Technology, University of Science and Technology Beijing, Beijing, 100083 China

**Keywords:** Microbiome, Microbial ecology, Soil microbiology

## Abstract

The process of microbiologically influenced corrosion (MIC) in soils has received widespread attention. Herein, long-term outdoor soil burial experiments were conducted to elucidate the community composition and functional interaction of soil microorganisms associated with metal corrosion. The results indicated that iron-oxidizing (e.g., *Gallionella*), nitrifying (e.g., *Nitrospira*), and denitrifying (e.g., *Hydrogenophaga*) microorganisms were significantly enriched in response to metal corrosion and were positively correlated with the metal mass loss. Corrosion process may promote the preferential growth of the abundant microbes. The functional annotation revealed that the metabolic processes of nitrogen cycling and electron transfer pathways were strengthened, and also that the corrosion of metals in soil was closely associated with the biogeochemical cycling of iron and nitrogen elements and extracellular electron transfer. Niche disturbance of microbial communities induced by the buried metals facilitated the synergetic effect of the major MIC participants. The co-occurrence network analysis suggested possible niche correlations among corrosion related bioindicators.

## Introduction

Engineering materials that are partially or completely buried in the soil are widely used in different industrial facilities^1,2^. The serviceability of these infrastructures is significantly affected by soil corrosion. Corrosion involves chemical interaction between the metal and its surrounding environment^3,4^. Microbes, as ubiquitous components of the soil environment, may participate in the corrosion process and alter corrosion behavior. The process is commonly known as microbiologically influenced corrosion (MIC) or biocorrosion.

In MIC studies, the most common corrosive microorganisms were observed to be sulfate-reducing bacteria (SRB), sulfur-oxidizing bacteria, iron-oxidizing bacteria (IOB), iron-reducing bacteria, acid-producing bacteria, and nitrate-reducing bacteria (NRB)^5^. The mechanism underlying the participation of these microorganisms in corrosion is often closely related to the elemental cycle of the environment^6,7^ and the abundance of microorganisms in biofilms formed on corroded metals^8^. For example, microaerophilic IOB play important biogeochemical roles along oxygen gradients in neutral pH and high Fe environments, and its corrosion products under anoxic conditions may further serve as substrates utilized by SRB^9^. The known IOB *Gallionella* and *Sideroxydans* were observed to be early colonizers on steel surface in a coastal marine environment and became the dominant species in the corresponding MIC community^10^.

Metal corrosion involves the interaction between metal materials and the surrounding environmental factors and microbes and is also accompanied by a series of chemical reactions and the formation of corrosion products. In the process of soil corrosion, metals are dissolved and lose electrons that are captured by suitable electron acceptors such as SO_4_^2−^, NO_3_^−^, O_2_, and surrounding microorganisms^11^. The consequent increase in cation concentration in the local soil environment may alter the soil ionic composition and its physicochemical properties, such as pH and electrical conductivity, and exert a deterministic effect on the microbial community^12^. Considering the large scale and diversity of underground metals and the long duration for which these are employed, the impact of metal corrosion may substantially alter the soil chemistry and microbial communities. The underestimation of these changes could lead to the misinterpretation of corrosion mechanisms and erroneous prediction of corrosion behavior of engineering structures in soil. Furthermore, understanding the interaction between corroding metals and the surrounding soil microbial community is also helpful in identifying the dominant corrosive microbes in soils and designing more meaningful experiments for MIC investigation. To the best of our knowledge, studies have reported the changes of microbial communities that occurred in the ecosystems under the influence of metal corrosion^13,14^, but the relationship between the changes of chemical factors caused by metal corrosion and surrounding microbes remain largely unexplored. The process by which the taxonomic and functional attributes of microbial communities are affected by the environmental variation governed by the corrosion process remains unknown^13,14^.

To study the interaction between the soil microbiome and metals during the corrosion process, burial tests were conducted in natural soil environments using different engineering materials, including a widely used carbon steel variant (Q235), a copper-bearing pipeline steel (X80Cu), and a polyethylene (PE) control, which is commonly used in inert protective coatings for underground pipelines. After 5 and 10 months, the buried materials and the surrounding soil samples were retrieved. The extent of corrosion was evaluated in terms of mass loss, surface morphology, and corrosion product formation. The surrounding soil microbial communities were studied using high-throughput sequencing. Comparative analysis of the microbial diversity and community assembly between soil samples collected at 2 and 10 cm horizontal distance from the materials was conducted. The major environmental determinants of ecological distribution of prokaryotic taxa were identified. The bioindicators of Q235 and X80Cu corrosion were recognized using random forest modeling, and the potential metabolic pathways associated with the corrosion process were determined through metagenomics prediction. Two co-occurrence microbial networks were constructed to elucidate the interaction between the corrosion-inducing microorganisms and to determine the possible mechanism underlying corrosion in the soil environment.

## Results

### Corrosion characterization

**Fig. 1:**
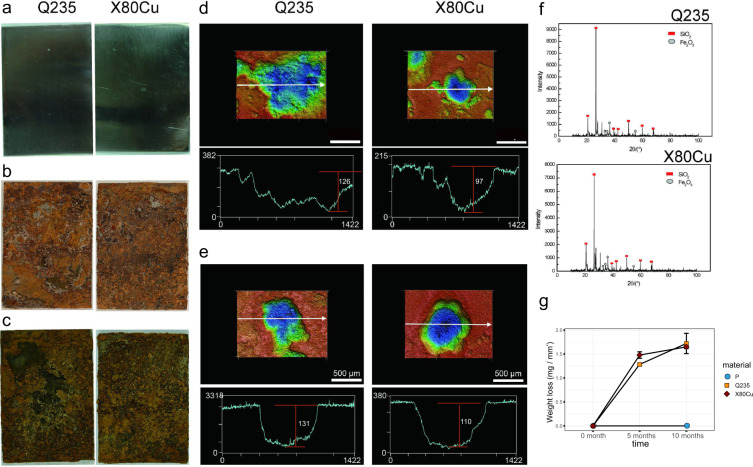
Corrosion observations of buried materials. Surface morphology of metal samples before burial tests (**a**), after 5 months (**b**), and after 10 months (**c**) of burial tests. Confocal laser scanning microscopic images of the corrosion depths after 5 months (**d**) and after 10 months (**e**) of burial tests; scale bar represented 500 μm. XRD analysis of crystalline phases of the corrosion products on the metal surfaces (**f**). Mass loss of materials (**g**).

After burial for only 5 months in the soil, severe corrosion occurred on the surface of Q235 and X80Cu steel coupons (Fig. 1). Both Q235 and X80Cu were covered by a thick layer of loose corrosion products. The Q235 coupon underwent a mass loss of 1.36 ± 0.04 g with a corrosion rate of 0.27 ± 0.02 g mm^−2^ month^−1^ and the maximum pit depth was ~126 μm (Fig. 1d, g). The mass loss of the X80Cu coupon was 1.57 ± 0.14 g, which corresponded to a corrosion rate of 0.33 ± 0.02 g mm^−2^ month^−1^ (Fig. 1g). The maximum depth of the corrosion pit was ~97 μm (Fig. 1d). After burial for 10 months, the pits on the surfaces of Q235 and X80Cu were further deepened. The maximum pit depths of Q235 and X80Cu were ~131 and ~110 μm, respectively (Fig. 1e). The mass loss of Q235 was 1.82 ± 0.44 g with a corrosion rate of 0.18 ± 0.04 g mm^−2^ month^−1^. The mass loss in X80Cu was 1.75 ± 0.07 g with a corrosion rate of 0.17 ± 0.007 g mm^−2^ month^−1^ (Fig. 1g). X-ray diffraction (XRD) analysis revealed that the corrosion products formed on the surfaces of Q235 and X80Cu had similar compositions and Fe_2_O_3_ was identified as the major crystalline phase component (Fig. 1f).

### Soil physiochemical properties

**Fig. 2:**
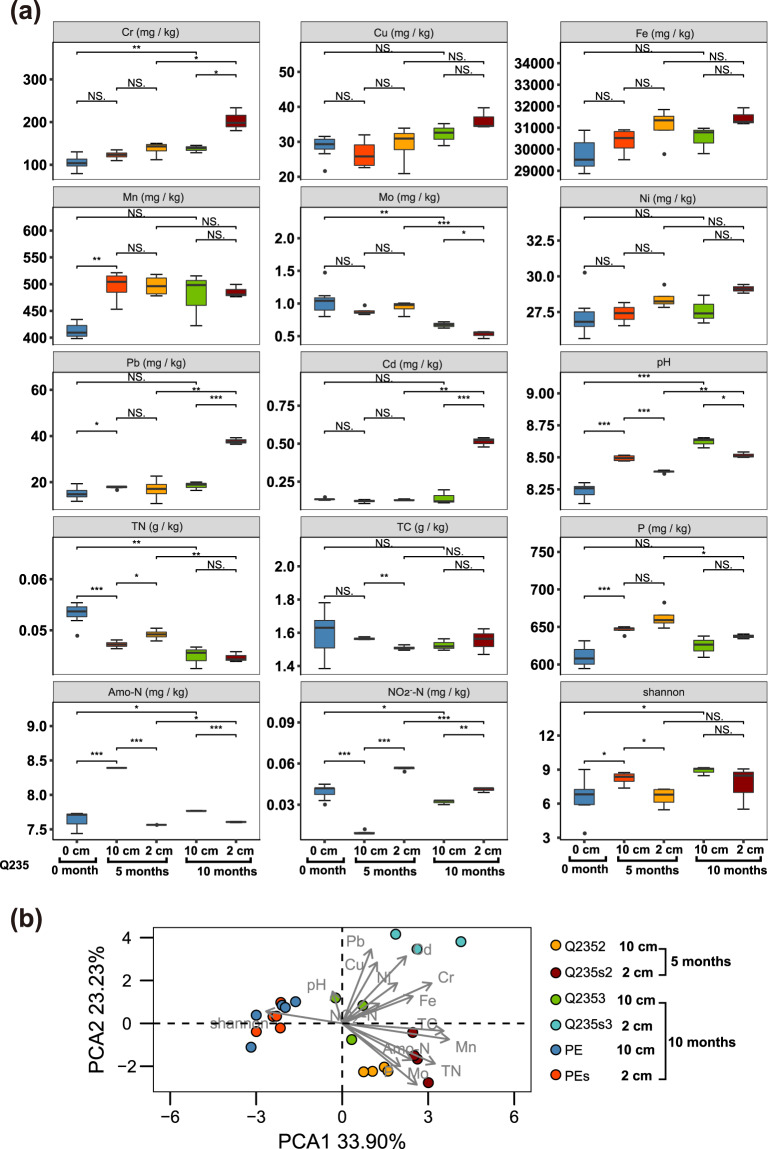
Physicochemical properties of the soil for Q235 groups. Differences in physicochemical properties of the soil at different time points and different distances (from left to right, *n* = 7, 4, 4, 3, and 3) for Q235 coupons (**a**) and principal component analysis (PCA) of soil parameters for Q235 and PE (**b**). Note: error bars represented SD; *p-value < 0.05; NS, means *p*-value > 0.05.

**Fig. 3:**
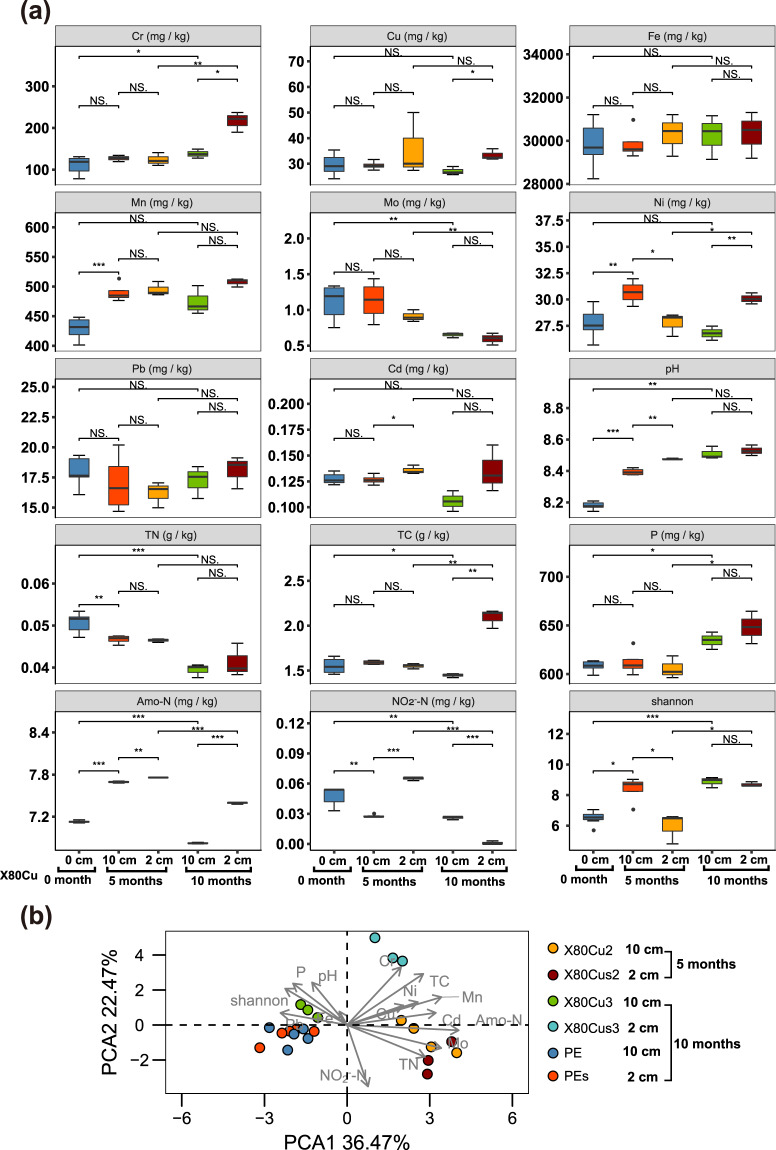
Physicochemical properties of the soil for X80Cu groups. Differences in physicochemical properties of the soil at different time points and different distances (from left to right, *n* = 7, 4, 3, 3, and 3) for X80Cu coupons (**a**) and principal component analysis (PCA) of soil parameters for X80Cu and PE (**b**). Note: error bars represented SD; *p-value < 0.05; NS, means *p*-value > 0.05.

The important soil parameters before and after coupons buried are listed in Supplementary Table 1. We performed principal component analysis (PCA) to estimate the impact of corroding metals on the physiochemical parameters of the soil by comparing the metal and PE groups (Figs. 2b and 3b). The total interpretation of the first two axes in the PCA plot for Q235 and PE was 57.13%. The contributors among the soil parameters were Cr, Fe, Mn, total nitrogen (TN), total carbon (TC), and Cr contents (Supplementary Fig. 1). The total interpretation of the first two axes in the PCA plot for X80Cu and PE was 58.94%. The major contributors among the soil parameters were Cr, Mn, Cd, TN, nitrite-N (NO_2_^−^-N), and ammonium-N (Amo-N) contents (Supplementary Fig. 2). Pairwise analysis of variance (ADONIS) revealed that soil samples at a horizontal distance of 2 cm from the metals were significantly different with those at a horizontal distance of 10 cm after 5 months (for Q235, F = 3.1, *p* = 0.02; for X80Cu, F = 2.7, *p* = 0.05) (Supplementary Tables 2 and 3). There was no significant difference between soil samples at 2 and 10 cm from the PE surface after 10 months (ADONIS, F = 0.69, *p* = 0.594) (Fig. 2 and Supplementary Table 4). Based on the statistical analysis of all the samples, it was shown that the horizontal distance from the corroding metals was a determinant factor that influenced the physiochemical properties of the soil (Figs. 2 and 3, and Supplementary Fig. 3).

### Diversity and composition variation of microbial communities

The Shannon index clearly indicated that the microbial diversity in soil within a horizontal distance of 10 cm from all material surfaces buried for 5 and 10 months was higher than that in the original soil environment according to Wilcoxon’s rank-sum test (wilcox test, *p* < 0.05), whereas the diversity of soil microbes at a horizontal distance of 2 cm from both metal surfaces buried for 5 months declined sharply in comparison to that at a horizontal distance of 10 cm (wilcox test, *p* < 0.05) (Figs. 2a and 3a, and Supplementary 3a).

**Fig. 4:**
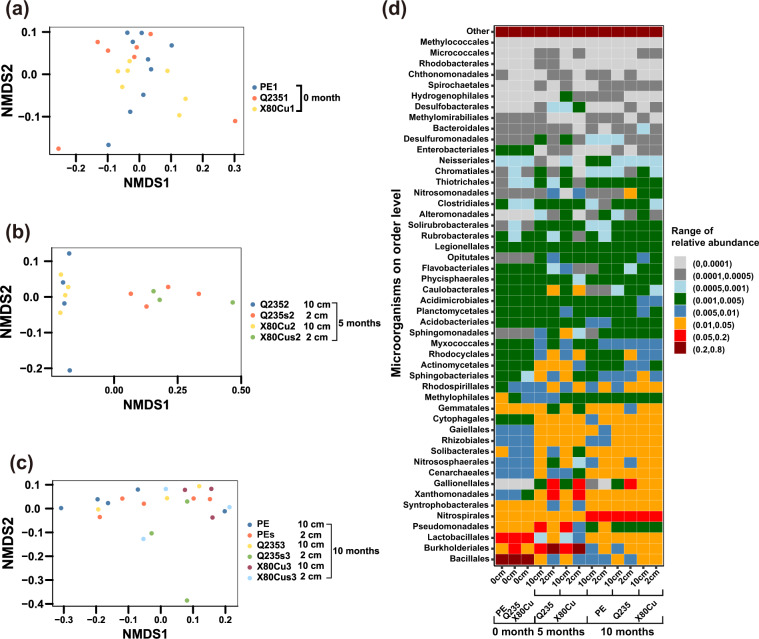
Differences in community composition of different materials. Non-metric multidimensional scaling (NMDS) analysis based on Bray–Curtis distance matrix among sample groups at different sampling time (**a**–**c**) and relative abundance of microbial orders detected in different soils (**d**).

Non-metric multidimensional scaling (NMDS) was performed using Bray–Curtis distances to analyze the discrepancy of microbial community structure among sample groups at different sampling times (Fig. 4a–c). The results showed consistency with diversity, i.e., samples at a horizontal distance of 2 cm from the metal surface buried for 5 months were observably different from those at 10 cm from the metal surface with respect to microbial community composition (for Q235, ADONIS, F = 3.73, *p* = 0.05; for X80Cu, ADONIS, F = 4.65, *p* = 0.05) (Supplementary Table 5). No significant difference existed between soil at 2 cm from Q235 and X80Cu surfaces buried for 5 months. The variance analysis results with respect to MDS1-MDS4 further confirmed the findings of the NMDS analysis and the significant differences between samples at 2 and 10 cm from the metal surface buried for 5 months (Supplementary Fig. 4). However, after 10 months, the difference between the microbial community structures in soil at 2 and 10 cm from the metal surface was no longer significant (for Q235, ADONIS, F = 1.17, *p* = 0.32, and for X80Cu, ADONIS, F = 0.94, *p* = 0.32) (Supplementary Table 6).

**Fig. 5:**
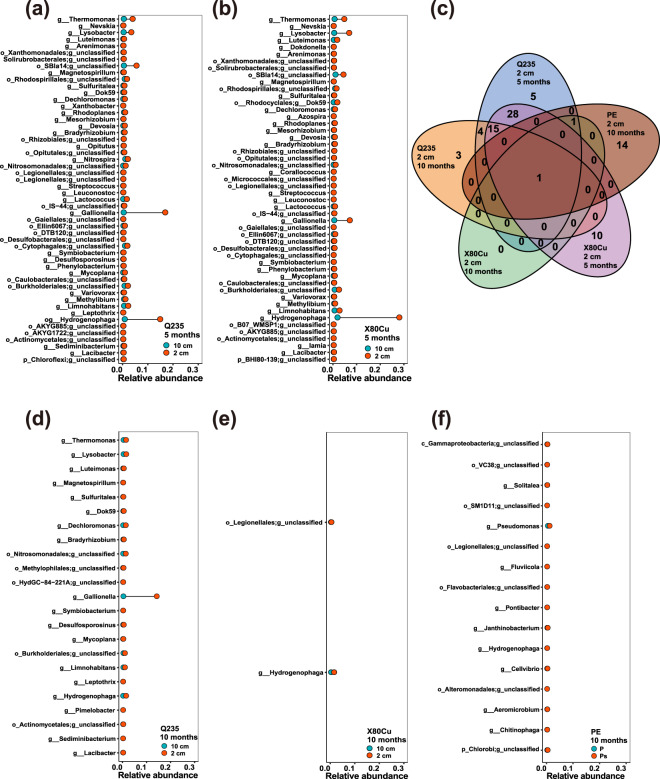
Taxa with relative abundance significantly increased in soil samples at 2 cm distance compared to that of 10 cm at the genus level. Distance from Q235 coupons at 5 months (**a**). Distance from Q235 coupons at 10 months (**b**). Distance from Q235 coupons at 5 months (**d**). Distance from Q235 coupons at 10 months (**e**). Distance from polyethylene at 10 months (**f**). Venn diagram of Taxa with increased abundance in different soil samples (**c**).

**Fig. 6:**
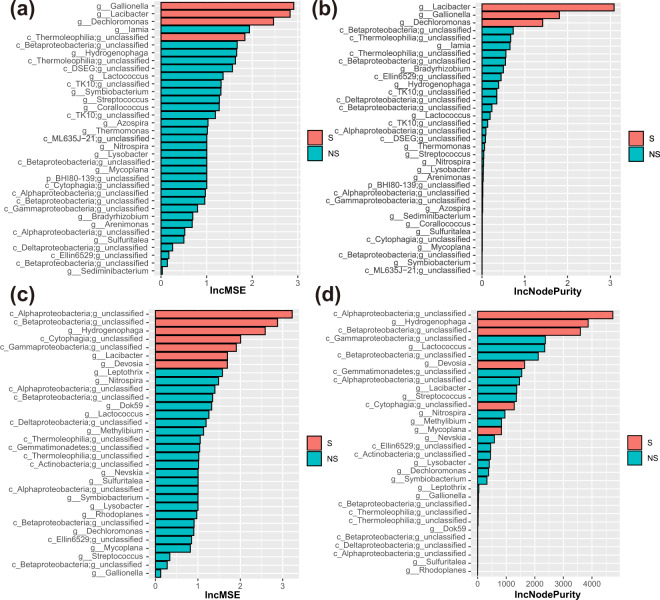
Representative taxa that responded positively to metal corrosion identified by Random Forest. The Increase in Mean Decrease Accuracy (IncMSE) (**a**) and Increase in Node Purity (IncNodePurity) (**b**) of random forest model calculated with the relative abundance of enriched microorganisms and metal mass loss data, IncMSE (**c**) and IncNodePurity (**d**) of random forest model calculated with the relative abundance of enriched microorganisms and redox potential data. Note, S means *p*-value < 0.05; NS means *p*-value > 0.05.

To identify the representative taxa that responded positively to metal corrosion, we focused on microorganisms at the class level (Fig. 4d) and at the genus level that had a significantly higher abundance in samples collected at 2 cm from the metal surface compared to those collected at 10 cm horizontal distance (Fig. 5). A total number of 81 genera showed significant high abundances, of which 44 were extremly abundant at 2 cm samples from both Q235 and X80Cu steels after 5 months (included *Gallionella*). After 10 months, 23 genera were still abundant at 2 cm samples from Q235 steel (included *Gallionella*), whereas only 2 genera were abundant in 2 cm samples from X80Cu (Fig. 5c). *Gallionella* and *Hydrogenophaga* had the highest relative abundance in samples collected at 2 cm from both metals buried for 5 months (Fig. 5a, b and Supplementary Table 7). The relative abundance of *Gallionella* in samples collected at 2 cm from Q235 continued to be the highest even after 10 months burial (Fig. 5d). Furthermore, the results in Fig. 6 showed that *Gallionella*, *Lacibacter*, and *Dechloromonas* were the most essential microbes (*p* < 0.05 for both IncMSE and IncNodePurity value), which potentially facilitated metal mass loss, whereas *Hydrogenophaga*, *Devosia*, an unclassified genus of *Betaproteobacteria*, and an unclassified genus of *Alphaproteobacteria* showed positive relationships with the variation of redox potential (Fig. 6).

### Relationship between environmental factors and microbial community structure

**Fig. 7:**
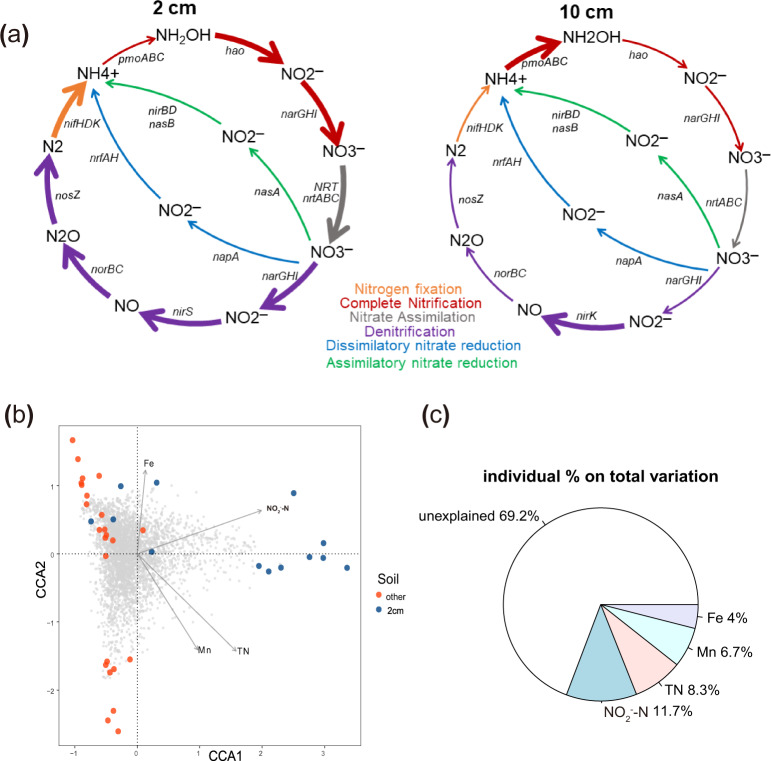
Correlation between environmental factors and microbial community and potential functions of microbiomes. Schematic diagram of nitrogen metabolism (**a**), canonical correlation analysis (CCA) of the microbial community (**b**), and variation partition of different variables (**c**) on microbial community dissimilarity of soil 2cm/10cm distance from metal coupons.

Canonical correlation analysis (CCA) was performed to identify the environmental factors that contributed most significantly to the variation of microbial community structure. The analysis indicated that there was a significant correlation between environmental factors and the microbial community distance matrix (*r* = 0.323, *p* = 0.001). Filtering of the environmental factors (all factors listed in Supplementary Table 1) showed that NO_2_^−^-N, TN, Mn, and Fe were significant contributing factors to the differences between microbial communities in samples collected at 2 and 10 cm horizontal distance from the metal surface. Among them, NO_2_^−^-N accounted for the highest interpretation of 11.7% (F = 6.305, *p* = 0.001), followed by TN (8.3%, F = 3.07, *p* = 0.001). Mn and Fe ions accounted for 6.7% (F = 3.224, *p* = 0.002) and 4% (F = 1.62, *p* = 0.05), respectively, of the difference in microbial community structure between samples from the 2 and 10 cm layers (Fig. 7b).

### Relationship between microbial phylotypes and metal correlation

**Fig. 8:**
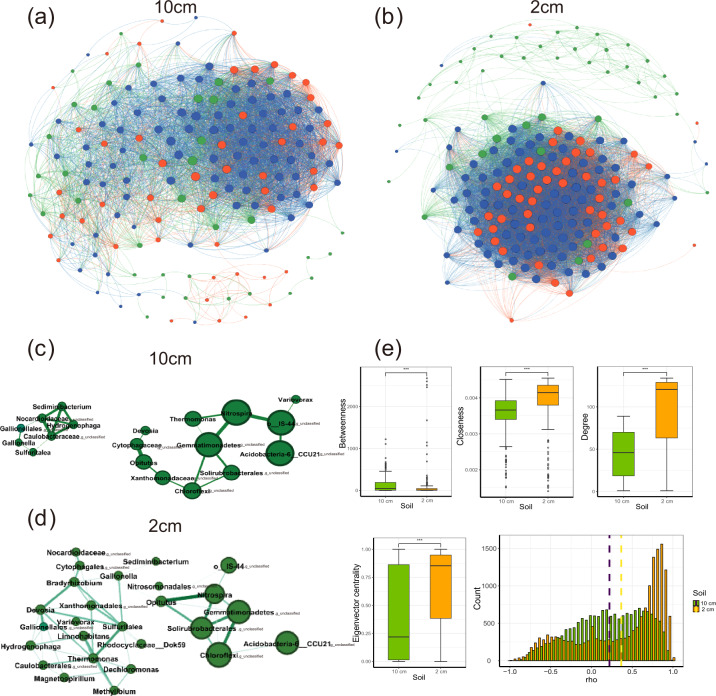
Microbial co-occurrence networks and its characteristics. Co-occurrence network analysis of the microbial communities (at genus level) of soil at 2 cm (**a**) and 10cm (**b**) distance from metal coupons. Taxa with the positive response to metal corrosion in soil 2 cm (**c**) and 10 cm (**d**) distance from metal coupons. Network characteristics of microbial co-occurrence network (from the left to right, *n* = 16 and 22) (**e**). The node size represented the node degree and the colors of nodes indicated the microbial response to metal corrosion in the network: green nodes, microorganisms with significantly higher relative abundance at 2 cm (positive response to metal); orange nodes, microorganisms of which relative abundance decreased significantly at 2 cm (negative response to metal); blue nodes, microorganisms of which relative abundance had no significant difference between 2 and 10 cm. Note, error bars represented SD; ***p-value < 0.0001.

To determine whether the presence of microorganisms and metal corrosion exert mutual influence, we constructed two microbial community co-occurrence networks using soil samples collected at 2 and 10 cm horizontal distance from the metal surface, respectively. The network for the samples collected at 2 cm included 177 nodes and 8359 edges, whereas that for samples collected at 10 cm had 170 nodes and 4152 edges (Supplementary Tables 10 and 11). Compared to the microbial communities in samples collected at 10 cm horizontal distance from the metal coupons (average correlation coefficients *r* were 0.22, *p* < 0.0001), those in samples collected at 2 cm horizontal distance had a greater number of positive correlation microorganisms (average correlation coefficients *r* were 0.36; *p* < 0.0001) (Fig. 8e). The betweenness of the 2 cm samples was lower than that of the 10 cm samples (2 cm, 124.1; 10 cm, 141.8; *p* < 0.0001). However, the node degree (2 cm, 93.0; 10 cm, 44.8; *p* < 0.0001), closeness (2 cm, 0.0037; 10 cm, 0.0035; *p* < 0.0001), and eigenvector centrality were higher in the network for the 2 cm samples (2 cm, 0.65; 10 cm, 0.40; *p* < 0.0001) (Fig. 8e). Statistical analysis of network characteristics revealed that there was a significant difference in microbial correlations between microbial communities corresponding to the 2 and 10 cm samples. Comparison of the network topological features indicated that the 2 cm microbial network had greater connectedness than the 10 cm microbial network and its nodes had closer relationships. Results of the microbial co-occurrence network suggested that microbial phylotypes responded variably toward metal mass loss.

To discern the major microbial contributors and the possible biotic interactions that contributed to the MIC of soil in our experiment, we focused on the important taxa identified by random forest analysis. Two smaller yet more distinct co-occurrence networks were formed and composed of 19 (for communities at a horizontal distance of 10 cm) and 27 (for communities at a horizontal distance of 2 cm) closely related genera, respectively. The larger node size and the thicker edges between the nodes suggest that the microbes were highly abundant and might have had stronger interactions. The nodes, which were enriched in the corrosion-impacted condition and significantly contributed to the mass loss, were majorly divided into three groups (Figs. 6 and 8, and Supplementary Table 12). The first group was present in the central part of both networks and had greater node degree and closeness. This group included *Nitrospira*, an unclassified genus of *Gemmatimonadetes*, an unclassified genus of *Solirubrobacterales*, and an unclassified genus of *Choloflexi* (Fig. 8c, d and Supplementary Table 12). The second group was located in the marginal area of the 2 cm network. The node degrees were lower and only displayed the internal correlation between group members. This category included *Gallionella*, *Bradyrhizobium*, *Dechloromonas*, *Hydrogenophaga*, *Sulfuritalea*, and *Magnetospirillum* (Fig. 8c, d and Supplementary Table 12). The third type of nodes held different niches between the networks for 2- and 10-cm soil samples. Microorganisms in this group included *Opitutus*, *Thermomonas*, an unclassified genus of *Cytophagales*, *Variovorax*, an unclassified genus of *Xanthomonadales*, and *Devosia*, respectively. (Fig. 8c, d and Supplementary Table 12).

### Functional and interaction studies of soil microorganisms associated with corrosion

In addition, the abundance of genes associated with important biogeochemical processes were analyzed using Picrust prediction. We observed that the genes related to nitrification (*hao*, *narGHI*), nitrogen fixation (*nifHDK*), nitrate assimilation *(NRT*), and denitrification (*narGHI, nirS*, *norBC*, *nosZ*) by microorganisms in samples collected at 2 cm horizontal distance from the metal surface were significantly enriched, which indicated the enriched metabolitic activities of nitrogen-cycling microorganisms (Fig. 7a). In addition, the gene cluster for sulfur oxidation (*soxABCXYZ*) and assimilatory sulfate reduction (*cysNDCHJI*) were significantly enriched in the 2 cm samples from both types of metals (Supplementary Fig. 5). With respect to heavy metal resistance and iron cycle, the genes for Mo resistance (*modADE*), Cu resistance (*cusB*, *pcoB*), Co/Zn/Cd resistance (*czcD*), Fe(III) uptake (*afuC*), and Fe(II) transport were significantly enriched in the 2 cm samples (Supplementary Fig. 6). In addition, there was a higher abundance of the genes encoding PilJ, PilI, PilH, and PilG that participate in biofilm formation, and CyaB and Vfr that are involved in the cAmp/Vfr signaling pathway, as well as of the gene cluster for the Type II secretion system (*gsp DEFGNM*) in soil at 2 cm horizontal distance from Q235 and X80Cu coupons buried for 5 months and from Q235 coupons buried for 10 months (Supplementary Fig. 7). Genes encoding proteins associated with the quorum sensing regulating system (*lasI/lasR*) and Psl polysaccharide biosynthesis (*algA*) also had significantly higher abundance in 2-cm samples than in 10-cm samples (Supplementary Fig. 7). For the electron transfer system, there was a higher abundance of gene clusters encoding the terminal electron acceptor, cytochrome c oxidase cbb3-type (*ccN, ccO, ccP, ccQ, ccNO*), and cytochrome bd complex (*cydA* and *cydB*) in samples collected at 2 cm horizontal distance from both types of metals buried for 5 months (Supplementary Fig. 8).

## Discussion

Owing to the significant economic loss and social impact associated with corrosion, intensive efforts have been made to elucidate the mechanisms underlying corrosion and to prevent the occurrence of corrosion. However, the influence of corroding metals on the surrounding environment have often been overlooked^15^. A particular study revealed that Fe and heavy metal ions such as Cr, Pb, and Sr could leach into the soil ecosystem as a result of external corrosion on buried pipes^4^. Our results indicated that corrosion of Q235 and X80Cu steels significantly increased the variances of metal ions concentration of Cr, Pb, and Cd in their surrounding soil environment. The increase in contents of Cr and Pb primarily attributed to ion dissolution during the corrosion process, which also reported by Sun et al.^16^.

The diversity and community structure, as well as the function of soil microorganisms, were significantly affected by the temporal variations of soil physiochemical properties and the effects of corroding metals. In our study, natural factors, primarily temperature, dissolved oxygen, and water content increment may have exerted positive effects on microbial diversity, as indicated by the Shannon indices. However, metal corrosion affected the biodiversity negatively. The release of metal ions upon soil corrosion reduced the microbial diversity and altered the microbial community structure in the surrounding soil environment^17^. Copper-bearing pipeline steel enriched less taxa at 2 cm distance for X80Cu compared to Q235 steels after 10 months. This might be due to the inhibitory effect of copper on microbial community in soil.

In a natural environment, microorganisms interact with each other through a complicated network. These biotic interactions could be either positive (e.g., mutualism, parasitism, and commensalism) or negative (e.g., competition and predation). Metal corrosion may become more severe owing to cooperative existence between corrosive taxa and may be inhibited owing to nutrition competition among microbial communities. The correlation networks of corrosion-related microbial taxa can provide substantial information about the biotic response toward the corrosion of buried metals and predict the genus that most significantly influences corrosion. Microbial co-occurrence analysis is a powerful method for visualizing and simplifying the intricate correlations among members of microbial communities. To discern the potential microbial interactions among the corrosion-related taxa or those that do not influence corrosion, we constructed two networks (based on microbial communities from samples collected at 2 and at 10 cm horizontal distance from the metal surface) using the microbial abundance profile. The microbial co-occurrence network around the corroding metal (at 2 cm horizontal distance from metal surface) showed higher node degree, eigenvector centrality, and closeness, which indicated greater correlation between soil microorganisms under the influence of corrosion^18,19^. The increased proportion of highly positive correlations in the network around corroding metals indicated that microbial community assembly was more unstable^20^. Furthermore, the synergetic effects of the microorganisms were stronger during the corrosion process. More intricate and dense biotic interactions and a reduction in biodiversity in a corrosion-affected environment suggested the deterministic influence of corroding metal on soil microbial assembly. In the early stages of corrosion (the first 5 months after metal burial in our experiment), the native microbial community was substantially altered with the enrichment of specific species under selective pressure, which increased the interspecific competition and reduced soil biodiversity. In later stages (10 months after metal burial in our experiment), the biodiversity and microbial assemblages were recovered, which suggested that the driving force of the corroding metal on microbial assemblages was weakened after 10 months. This possibly resulted from oxygen depletion and biofilm formation on the metal surface.

In the co-occurrence network, the microbes that respond positively to metal corrosion were classified into three types. At the core of the network, a group of microbial taxa principally containing *Nitrospira*, an unclassified genus of *Gemmatimonadetes*, an unclassified genus of *Acidobacteria*, an unclassified genus of *Solirubrobacterales*, and an unclassified genus of *Choloflexi* were closely related to other nodes. These microorganisms had high node degrees in both networks with or without the corrosion effect, which indicated that they played an essential role in affecting other microorganisms during the corrosion process. These microbes also encountered lower environmental stress from the corroding metals. *Acidobacteria* and *Nitrospira* have been considered to follow a distinct life cycle strategy and are more competitive in harsh environments^21^. *Nitrospira, Gemmatimonadetes*, and *Choloflexi* were previously identified as members of core microbiome and keystone taxa in soil^22^, which is also consistent with our results. *Nitrospira*, characterized as nitrifying bacteria that can oxidize nitrite to nitrate. *Nitrospira* has been reported to harbor a complete set of *amo* and *hao* genes, which may enable it to oxidize both NO_2_^−^ and NH_4_^+^ to NO_3_^−^ and perform complete ammonia oxidation^23^. In addition, the increase in nitrate nitrogen may have induced the enrichment of microorganisms related to denitrification and nitrate assimilation, such as the closely related node *Opitutus* depicted in the 2 cm co-occurrence network (Fig. 8c, d).

A different group of corrosion-related microbes was located in the marginal area of the network in soil samples collected at 2 cm horizontal distance (Fig. 8c, d). The microbial taxa in this category primarily included *Gallionella*, *Bradyrhizobium*, *Dechloromonas*, *Hydrogenophaga*, *Sediminibacterium*, *Sulfuritalea*, and *Magnetospirillum*. There was limited correlation between the presence of microorganisms and core microorganisms in the network, which suggested that the niche of these microorganisms may not affect the major ecological functions of local microbiota. However, these microorganisms are considered conditional opportunistic species in the soil environment and occupy an advantageous position when the environment is suitable^24,25^. These microbes were significantly enriched in soil at 2 cm distance for both Q235 and X80Cu steels and seemed to be not particularly prone to Cu inhibition. The buried metal and initial dissolution of metal ions increased the concentration of nutrient elements, which led to the recruitment of IOB such as *Gallionella*, *Dechloromonas*, and *Sediminibacterium*, and increased the abundance of these microbes around the metal surface. Our results revealed that the average relative abundance of *Gallionella* in corrosion-unaffected soil samples was lower than 1%; however, it increased significantly in corrosion-affected soil (12%). *Gallionella* spp. are microaerobic IOB from the family *Gallionellaceae* and participate in iron oxidation in various aqueous environments^26,27^. Nitrification and zero-valent iron oxidation on the metal surface can reduce oxygen concentration and create a microaerobic environment around the corroding metal. Therefore, iron oxidation in soil corrosion may be majorly mediated by microaerobic IOB. In the presence of nitrates, *Gallionella* may produce the incompletely reduced substrate nitric oxide, which is toxic by itself. Therefore, they might collaborate with related microbia such as *Bradyrhizobium*, *Hydrogenophaga*, and *Magnetospirillum*, which follow a denitrification pathway to complete the nitrate reduction process^28^. This correlation was also indicated in the co-occurrence network in our study (Fig. 8). In addition, as nitrite could react abiotically with Fe(II) to form N_2_O with NO as an intermediate at near-neutral pH, if this reaction occurred, a large quantity of Fe(III) precipitate would be formed through Fe(II) oxidation by nitrite, which might explain the formation of a large quantity of rust around the corroding metal. The denitrifying iron-oxidizing *Betaproteobacteria Dechloromonas* has been considered an important taxon for nitrate-dependent Fe^2+^ oxidation and was observed to share a strong and significant correlation with Fe^3+^-reducing bacteria^29^.

The third type of microorganisms occupied different niches between the 2 and 10 cm networks. Nevertheless, these taxa exhibited a denser correlation with other corrosion-related microorganisms in the corrosion-affected network, which suggested that metal corrosion altered their niche, possibly owing to direct influence under environmental selective pressure or/and indirect biotic correlation with other corrosion-related taxa. The microorganisms in this category included *Opitutus*, *Thermomonas*, an unclassified genus of *Cytophagales*, an unclassified genus of *Xanthomonadales*, and *Devosia* (Fig. 8c, d). Similar to hydrogen autotrophic denitrifying bacteria *Hydrogenophaga*, *Opitutus* was confirmed to be capable of efficiently reducing NO_3_^−^ using hydrogen as an electron donor^30^. Increased NO_3_^−^ and hydrogen contents in a corrosion-impacted environment may serve as important factors that affect the niche and abundance of hydrogen autotrophic denitrifying bacteria *Hydrogenophaga* and *Opitutus*. In addition, denitrifying bacteria may be closely associated with nitrifying bacteria. This was suggested in the 2-cm co-occurrence network, where *Opitutus* shared a significantly stronger correlation with the dominant nitrate producer, *Nitrospira* (Fig. 8d). Unclassified genera of *Xanthomonadales* and the anoxic denitrifier *Thermomonas*, are believed to be able to excrete large quantities of extracellular polymeric substances and play an important role in biofilm development. Biofilms may further serve as a heterotrophic carbon source for other microorganisms. This may explain the existed of a dense correlation in the network of the two nodes between related taxa.

In this study, TN and NO_2_^−^-N contents were regarded as the most important environmental factors, which acted as the primary contributor to the variation of microbial community assembly at 2 and 10 cm horizontal distance from the metal surface. The enrichment of genes associated with nitrogen cycling was also observed in the 2 cm samples. It is generally acknowledged that SRB are the primary causative agents of MIC in marine and petroleum environments. However, in our study, SRB were not the dominant taxa in soil samples, and the genes responsible for dissimilatory sulfate reduction were non-abundant in corrosion-affected soil. However, the response of iron-oxidizing (primarily *Gallionella*), nitrifying (an unclassified genus of *Choloflexi* and *Nitrospira*), and denitrifying (*Hydrogenophaga*, *Magnetospirillum*, *Dechloromonas*, *Bradyihizobium*, and *Opitutus*) microorganisms to soil metal corrosion was observed, and genes associated with denitrification (*narGHI*, *nirS*, *norBC*, and *nosZ*) showed significantly higher abundance in corrosion-affected environments. NRB was previously believed to suppress SRB growth in oil and gas fields, thus inhibiting H_2_S production by SRB and reducing metal corrosion^31^. However, iron oxidation coupled with nitrate reduction can support the growth of NRB and further promote metal corrosion via extracellular electron transfer, as demonstrated in this study and previous report^32^. The characterization of environmental factors, microbial community structure, and taxa interaction analysis suggested that iron oxidization and nitrate cycling played important roles in MIC in sulfate-deficient soil environments. Denitrification coupled with iron oxidization may further drive the nitrogen biological cycle in the soil environment and help recruit microorganisms that exert synergetic effects. The presence of iron-cycling and nitrogen-cycling microbes and the correlations between these microorganisms suggested that microbiologically influenced metal corrosion may be dominated by microbially driven Fe-N redox cycling in a neutral soil environment. The metabolic interaction among microorganisms and electronic communication through extracellular electron transfer may form an important component of MIC in a neutral soil environment. Functional prediction of the microbial community by metagenome analysis revealed that the quorum sensing system, terminal electron acceptor system, and polysaccharide biosynthesis system were enhanced in corrosion-influenced samples, which indicated the enhanced extracellular electron transfer among microorganisms during the corrosion process.

Overall, our results reveal the inter-relationship between steels, microbes, and biogeochemical cycling in the soil environment. We developed a list of bioindicators that could predict the chances of Q235 and X80Cu corrosion in soil and extended our understanding of microbiological functions on metal corrosion. In addition, intricate interactions among the corrosion-related microorganisms were suggested and the possible electrochemical communication pathway via extracellular electron transfer was proposed. Further research is warranted to isolate pure strains to confirm their roles and the mechanisms underlying corrosion, as well as to focus on the quantification of matter cycles associated with iron or nitrogen, which could help develop a holistic idea of the relationship between MIC and the environment.

## Methods

### Sampling location and strategy

The Yangfang Corrosion Test Station (116°16′ E, 39°59′ N), Changping District, Beijing was selected for conducting the metal material corrosion experiments under natural conditions. This site had a typical warm temperate, sub-humid, continental monsoon climate. The average annual temperature was 11.8 °C and the average precipitation was 550–600 mm, concentrated in summer. A 4 m × 4 m square plot was selected and excavated to an approximate horizontal distance of 2 m from the ground. The materials used in this experiment were cut into 5 cm × 8 cm × 1 cm coupons. Prior to the long-term outdoor soil exposures, all the metal coupons were abraded, polished, cleaned, and weighed as described earlier^33^. Seven copies of each test material were used for biological reproduction.

19 sample points were set up at 50 cm intervals and marked as a1–a7, b1–b7, and c1–c5. The soil samples S1–S19 were collected at the corresponding positions of the sample points. Concurrently, the Q235 carbon steel coupons Q1–Q7 were placed vertically at positions of a1–a7, X80Cu pipeline steel coupons X1–X7 at b1–b7, and PE polymer materials at c1–c5. After the burial operation was completed, the upper layer of soil was buried back into the plot. After 5 months of experiment burial, the corresponding positions of the Q1–Q4 and X1–X4 samples were excavated. The metals were stored in place and samples were collected from soil at a horizontal distance of 2 cm from Q235 (named Qs2_1–Qs2_4) and X80Cu (named Xs2_1 –Xs2_4) surfaces, and at a horizontal distance of 10 cm from Q235 (named Qs10_1–Qs10_4) and X80Cu (named Xs10_1–Xs10_4) surfaces. Concurrently, temperature, moisture, and electrical conductivity values at each sampling point were measured immediately. Next, the buried materials were removed. After 10 months of experimental burial, the corresponding positions of Q5–Q7, X5–X7, and P1–P5 were excavated. The sampling method was the same as that mentioned above. Samples were collected from soil at a horizontal distance of 2 cm from Q235 (named Qs2_5–Qs2_7), X80Cu (named Xs2_5–Xs2_7), and PE (named Ps2_1–Ps2_5) surfaces; samples were also collected at a horizontal distance of 10 cm from Q235 (named Qs10_5–Qs10_7), X80Cu (named Xs10_5–Xs10_7), and PE (named Ps10_1–Ps10_5) surfaces. Eventually, 56 soil samples and 15 metal samples were obtained (Table 1). The test metals were weighed and the redox potential values were measured. Soil samples were collected in sterile plastic bags using a shovel and were transported to the laboratory within 4 h. DNA extractions for microbial analysis were completed within two days and the soil samples for physical and chemical characterization were stored at 4 °C until used.

**Table 1:** Samples information.

Samples	Group	Numbers	Time	Distance from material	Corresponding material
S1–S19	Soil	19	201606	None	None
Qs10_1–Qs10_4	Q2352	4	201611	10 cm	Q235
Xs10_1–Xs10_4	X80Cu2	4	201611	10 cm	X80Cu
Qs2_1–Qs2_4	Q235s2	4	201611	2 cm	Q235
Xs2_1–Xs2_3	X80Cus2	3	201611	2 cm	X80Cu
Qs10_5–Qs10_7	Q2353	3	201703	10 cm	Q235
Xs10_5–Xs10_7	X80Cu3	3	201703	10 cm	X80Cu
Qs2_5–Qs2_7	Q235s3	3	201703	2 cm	Q235
Xs2_5–Xs2_7	X80Cus3	3	201703	2 cm	X80Cu
Ps10_1–Ps10_5	PE	5	201703	10 cm	Polyethylene
Ps2_1–Ps2_5	PEs	5	201703	2 cm	Polyethylene

### Physical and chemical characterization of samples

Soil temperature, water content, and conductivity were measured in situ using a W.E.T. sensor (Eijkelkamp, Giesbeek, The Netherlands). For pH measurement, the soil samples were air-dried for at least 2 days and mixed with water (1:3, w/v). TC and TN were measured using elemental analysis system (Elemnetar Vario Max CN)^34^. For Amo-N and NO_2_^−^-N measurement, 5 g soil samples were mixed with 1 M KCl (1:3, w/v), vortexed thoroughly by shaker and then were measured using Thermo Scientific Aquakem 600^35^. The concentrations of metals including Fe, Cu, Cd, Ni, Mn, Mo, Cr, and Pb and the concentration of P were measured using inductively coupled plasma optical emission spectrometry (Optima 5300DV, PerkinElmer, USA) after crushing and sieving the soil samples using a 0.074 mm sieve^34^. The surface morphology of the metal coupons after 5 and 10 months was characterized using an ultra-deep digital microscope (KEYENCE, China), and the crystalline phases of the corrosion products scraped from the metal surface were analyzed using XRD analysis. The corrosion rates of different metals were determined according to the mass loss method and redox potential difference (Redox) of the material.

### Generation and processing of 16S rDNA amplicon sequences

Genomic DNA was extracted from 0.5 g of soil sample using the Omega Soil DNA Kit (Omega Bio-tek, Georgia, USA) according to the manufacturer’s instructions. The variable regions V4–V5 of the 16S rDNA genes were amplified using the universal primer set 515F (5′-GTGCCAGCMGCCGCGG-3′)/907R (5′-CCGTCAATTCMTTTRAGTTT-3′). Each PCR sample was prepared with 4 μL 5X FastPfu Buffer, 2 μL 2.5 mM dNTPs, 0.8 μL of each primer (5 μM), 0.2 μL bovine serum albumin, 0.4 μL FastPfu Polymerase, and 10 ng template DNA, and PCR-grade water was added up to 20 μL. PCR amplification was performed in triplicate and consisted of the following steps: denaturation for 3 min at 95 °C; 30 cycles of 30 s at 95 °C, 30 s at 55 °C, 45 s at 72 °C; extension for 10 min at 72 °C. The PCR products were separated by gel electrophoresis and sequenced using the Illumina 1.9 Miseq platform. Bioinformatic processing was performed using a combination of QIIME, USEARCH, UPARSE, and R^36–39^. The raw data were trimmed and quality-filtered using expected-error threshold < 1 and singleton-discarded using USEARCH, clustered into operational taxonomic units (OTUs) at 97% identity using UPARSE, and assigned taxonomy by comparison to the Greengenes 13_8 database using the Ribosomal Database Project classifier^40^. The OTU representative sequences were filtered, which included the removal of chimeric and chloroplast and mitochondrial phylotypes, and the results were aligned to generate the OTU table. At the final filtering stage, OTUs that were corresponded to less than 20 sequences in all samples were removed owing to the absence of biological interpretation^41^.

### Statistical analysis

To estimate the soil microbial diversity, the OTU table was normalized to the same number of reads per sample. PCA was conducted using soil environmental parameters and alpha diversity indices (Shannon indices)^42^. For NMDS analysis, the Bray–Curtis distance was calculated to compare the differences in microbial assembly among groups (using QIIME)^43^. Environmental variables, including the Shannon index, Cr, Cu, Fe, Mn, Mo, Ni, P, Pb, pH, TN, TC, Cd, Amo-N, and NO_2_^−^-N levels were considered for CCA analysis. Statistical differences of environmental variables among groups were calculated using wilcox test. The Mantel test was used to quantify the relationship between environmental factors and microbial assembly. The OTU counts were normalized using logarithmic transformation, and step modeling was used to test the lowest Akaike information criterion (AIC) value to filter unimportant factors. The Rdaenvpart package was used for the interpretation of each environmental factor. Differences in microbial composition between sample groups at the phylum, order, class, and genus levels were determined using the edgeR package, and significance was tested using ADONIS with 10,000 permutations. The random forest package of R statistical software was used to identify the microbial groups that were significantly correlated to the mass loss^44^. To evaluate the importance of each microbial indicator, two indices, increase in mean decrease accuracy (IncMSE) and increase in node purity (IncNodePurity) of random forest analysis were calculated when the data for that indicator was permuted randomly while others unchanged. Higher IncMSE and IncNodePurity values indicated that the variations in mass loss and redox potential were attributed to the more crucial microorganisms. *P*-values for multiple comparisons were adjusted using the false discovery rate using the Benjamini–Hochberg method. All statistical analyses were performed in an R environment.

### Co-occurrence network construction

Microbial co-occurrence networks were constructed to characterize the microbial niche at a horizontal distance of 2 and 10 cm from the metal surface. Correlation calculations were performed using the genera that appeared in at least five samples and had more than ten average sequences in the sample. Pairwise Spearman’s correlation was performed and correlations with coefficients < 0.6 or *P* > 0.05 were filtered^45^. The positive correlations were exclusively focused on based on their mathematical interpretation and biological meaning^46^. The global network properties were calculated to describe the topology using igraph packages in R^47^ and, the networks were visualized using Gephi.

### Reporting summary

Further information on research design is available in the Nature Research Reporting Summary linked to this article.

## Supplementary information

Supplementary Information

Reporting Summary

## Data Availability

The datasets generated for this study can be found in NCBI with accession code PRJNA642359.

## Code availability

Code and detailed information on the computing steps are available on github (https://github.com/Rheannna/Responses-of-soil-microbiome-to-steel-corrosion).
