# Supplementary material for: Responses of soil microbiome to steel corrosion
Source: NPJ Biofilms Microbiomes. 2021 Jan 21;7:6. doi: 10.1038/s41522-020-00175-3 (PMC7820017; doi:10.1038/s41522-020-00175-3)
Supplement: Supplementary file 1 — Supplementary Information [file 41522_2020_175_MOESM1_ESM.pdf]

Responses of soil microbiome to steel corrosion

Ye Huang[1, 2†], Dake Xu[3†], Lu-yao Huang[4], Yun-tian Lou[4], Jiang-Baota Muhadesi[1, 2], Hong-chang Qian[4], En-ze Zhou[3], Bao-jun Wang, Xiu-Tong Li[1, 2], Zhen Jiang[1, 2], Shuang-Jiang Liu[1, 2], Da-wei Zhang[4]*, Cheng-Ying Jiang[1, 2]**

[1]State Key Laboratory of Microbial Resources, Institute of Microbiology, Chinese Academy of Sciences, Beijing, 100101, P. R. China

[2]University of Chinese Academy of Sciences, Beijing 100049, P. R. China

[3]Shenyang National Laboratory for Material Sciences, Northeastern University, Shenyang 110819, China

[4]Beijing Advanced Innovation Center for Materials Genome Engineering, National Materials Corrosion and Protection Data Center, Institute for Advanced Materials and Technology, University of Science and Technology Beijing, Beijing 100083, China

[†]These authors contributed equally to this work.

Corresponding Authors

*E-mail: liusj@im.ac.cn (S.J. Liu)

*E-mail: dzhang@ustb.edu.cn (D.W. Zhang)

*E-mail: jiangcy@im.ac.cn (C.Y. Jiang)

Supplemental Figures

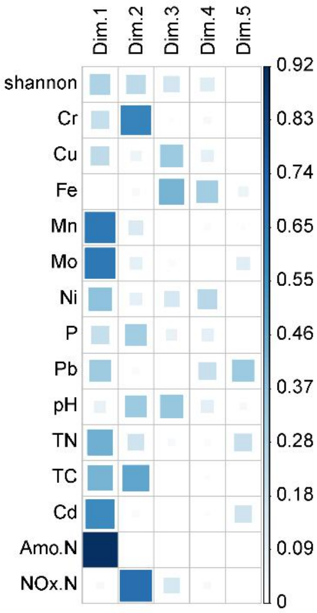

Supplementary Figure 1. Contributions of each soil parameters upon first five dimensions in PCA plot of X80Cu

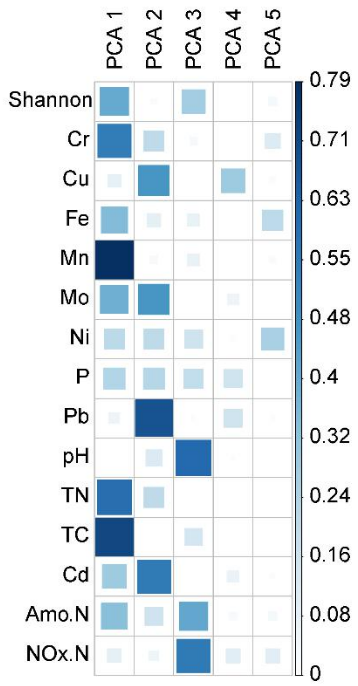

Supplementary Figure 2. Contributions of each soil parameters upon first five dimensions in PCA plot of Q235

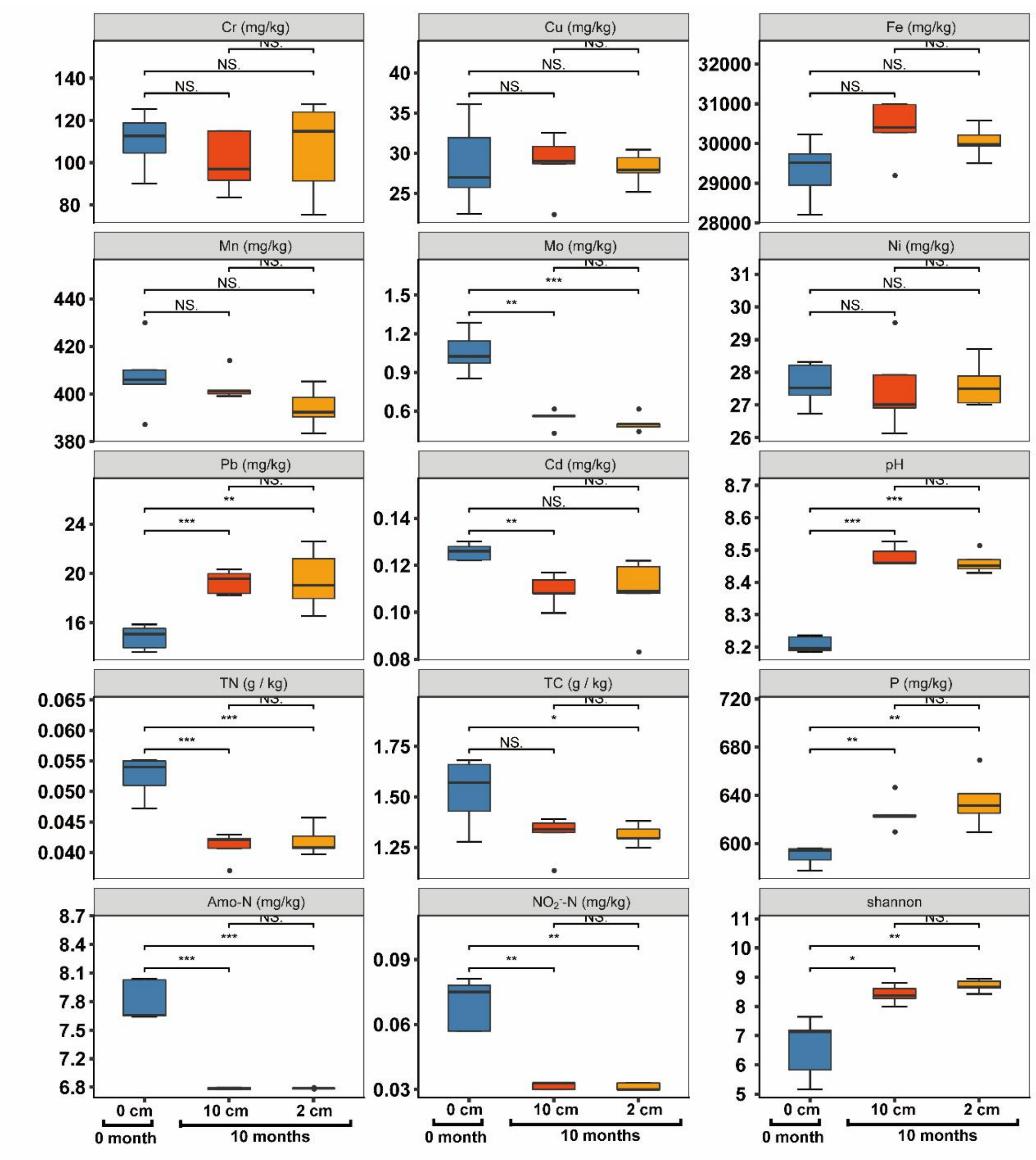

Supplementary Figure 3. Differences in physiochemical properties of the soil at different time points and at different distances for PE (from left to right, n = 5, 5, and 5). Note, Error bars represented SD; *, means p-value < 0.05; NS, means p-value < 0.05.

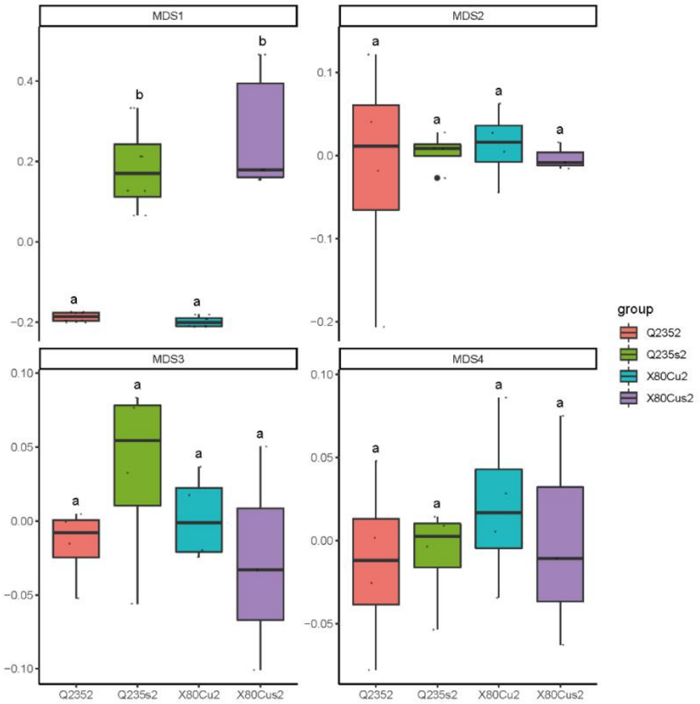

Supplementary Figure 4. Analysis of variance regarding the MDS1-MDS4 of NMDS analysis for sample at 2 cm and 10 cm distance from metals surface buried for 5 months.

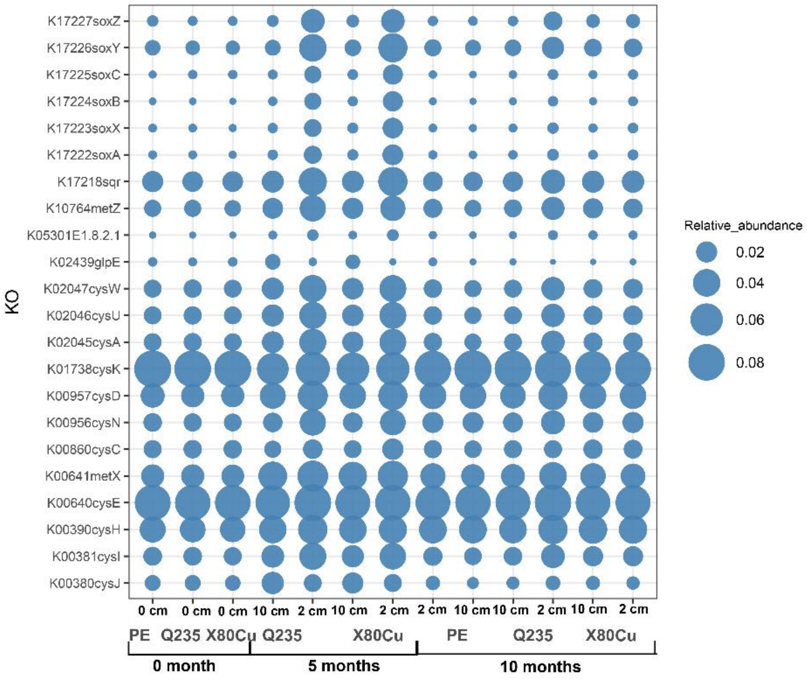

Supplementary Figure 5. Percentage of reads annotated as genes for sulfur metabolism for all groups of soil microbial communities. Genes shown in plot were significantly abundant in samples of 2 cm distance from metals compared with 10 cm samples. Note:

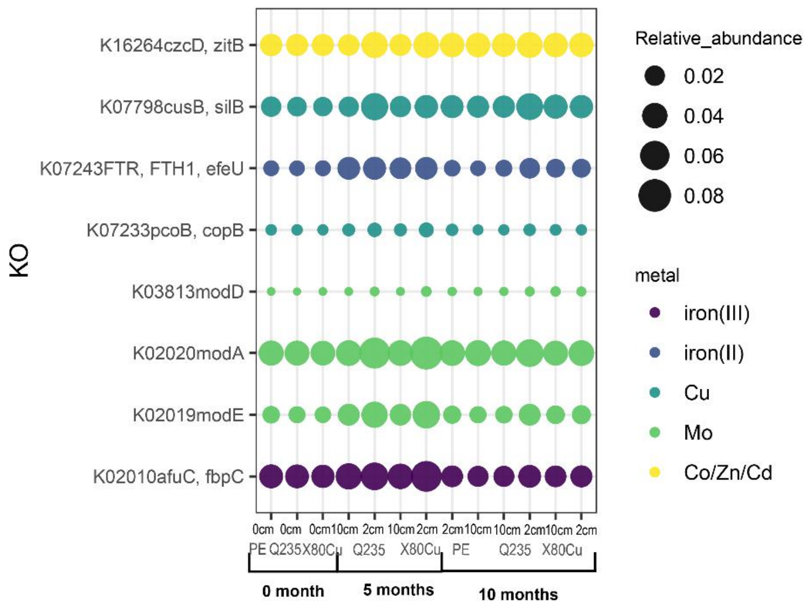

Supplementary Figure 6. Percentage of reads annotated as genes for metal resistance and iron uptake and transport for all groups of soil microbial communities. Genes shown in plot were significantly abundant in samples of 2 cm distance from metals compared with 10 cm samples.

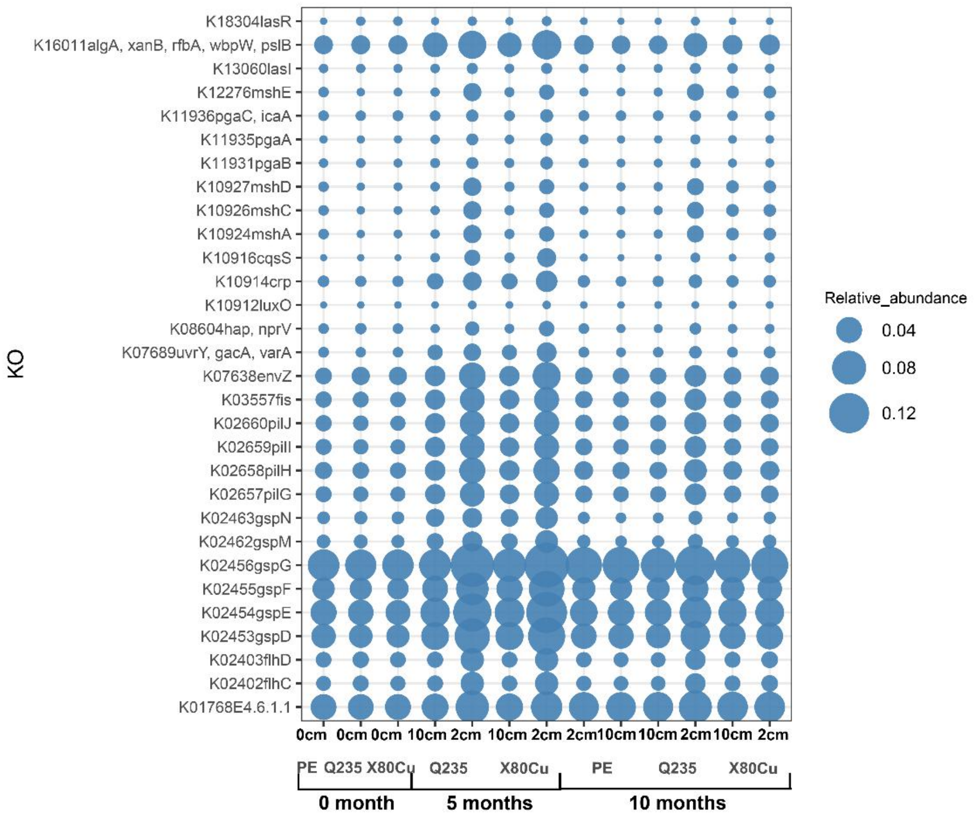

Supplementary Figure 7. Percentage of reads annotated as genes for biofilm formation for all groups of soil microbial communities. Genes shown in plot were significantly abundant in samples of 2 cm distance from metals compared with 10 cm samples.

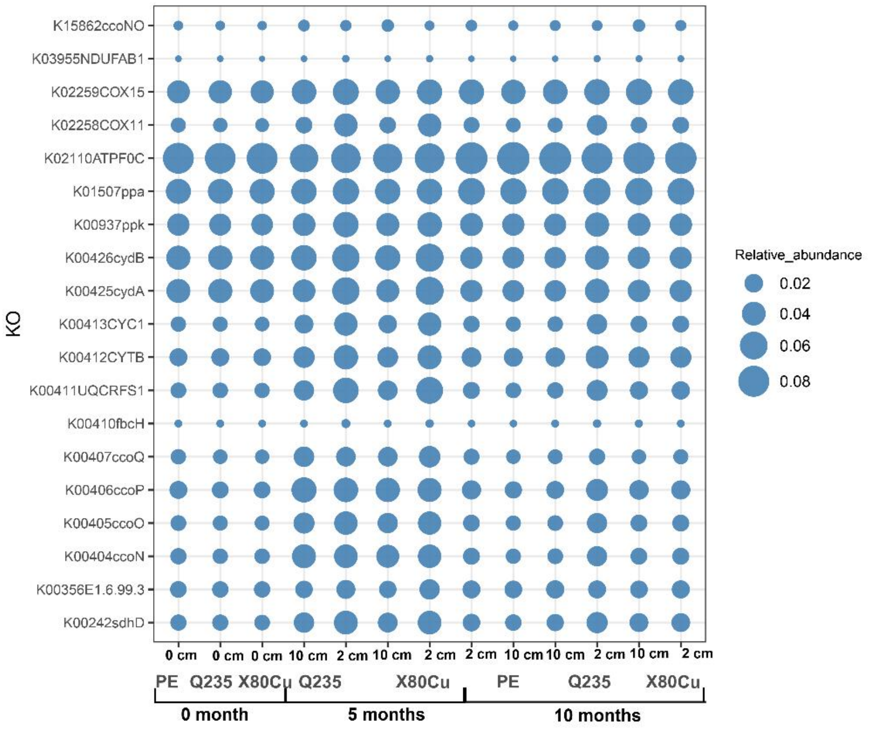

Supplementary Figure 8. Percentage of reads annotated as genes for electron transfer for all groups of soil microbial communities. Genes shown in plot were significantly abundant in samples of 2 cm distance from metals compared with 10 cm samples.

Supplemental Tables

Supplementary Table 1 Soil properties

sample ID	group	Cr (mg/kg)	Cu (mg/kg)	Fe (mg/kg)	Mn (mg/kg)	Mo (mg/kg)	Ni (mg/kg)	P (mg/kg)	Pb (mg/kg)	Temp (°C)	ECp (mv)
S15	P1	104.62	26.99	28950	404.10	1.28	27.30	595.50	13.62	33.90	209.10
S16	P1	112.67	25.75	29519	406.10	1.14	28.22	577.30	15.86	33.90	209.10
S17	P1	125.42	31.93	30227	429.90	0.97	27.52	586.50	13.98	33.90	209.10
S18	P1	90.12	22.47	29739	410.10	1.03	26.73	596.00	15.08	33.90	209.10
S19	P1	118.83	36.09	28208	387.10	0.85	28.32	594.10	15.54	33.90	209.10
S1	Q2351	99.96	29.20	29340	403.10	1.05	25.65	607.90	14.78	33.90	209.10
S2	Q2351	94.37	26.58	28871	415.70	1.47	26.81	600.27	16.45	33.90	209.10
S3	Q2351	110.39	21.58	29100	409.50	1.12	26.19	594.60	16.40	33.90	209.10
S4	Q2351	130.19	31.56	29520	429.80	0.92	27.27	615.70	19.34	33.90	209.10
S5	Q2351	79.31	31.39	30315	398.40	1.04	27.77	600.50	11.73	33.90	209.10
S6	Q2351	103.72	29.33	30884	402.60	0.80	30.25	631.50	13.23	33.90	209.10
S7	Q2351	116.05	30.09	30289	434.10	0.88	26.82	624.30	14.04	33.90	209.10
S8	X80Cu1	90.12	31.77	31211	442.30	0.90	27.56	604.30	17.46	33.90	209.10
S9	X80Cu1	110.39	33.10	30885	427.50	1.03	29.16	625.10	15.03	33.90	209.10
S10	X80Cu1	130.89	29.06	30696	445.10	1.32	26.79	608.60	18.80	33.90	209.10
S11	X80Cu1	127.81	24.13	29372	431.70	0.97	27.52	612.50	19.33	33.90	209.10
S12	X80Cu1	125.87	35.35	30472	426.10	1.33	29.80	612.60	16.08	33.90	209.10
S13	X80Cu1	103.72	27.02	29688	448.20	1.30	25.67	608.10	19.30	33.90	209.10
S14	X80Cu1	78.12	33.15	28241	411.60	1.19	27.46	613.50	17.62	33.90	209.10
Qs2_1	Q235s2	139.55	33.92	31845	478.10	1.01	28.33	657.70	10.74	19.60	199.70

Qs2_2	Q235s2	146.47	20.91	31428	483.20	1.00	27.83	682.30	16.40	19.60	199.70
Qs2_3	Q235s2	150.16	30.02	29769	518.30	0.80	29.41	648.50	22.64	19.60	199.70
Qs2_4	Q235s2	111.75	31.83	31261	509.10	0.96	28.16	660.40	17.81	19.60	199.70
Qs10_1	Q2352	125.12	23.48	30798	513.20	0.86	28.16	648.10	18.20	19.60	199.70
Qs10_2	Q2352	121.38	28.17	30243	521.30	0.86	27.14	648.10	18.24	19.60	199.70
Qs10_3	Q2352	134.87	31.99	29514	495.60	0.97	26.55	637.70	16.54	19.60	199.70
Qs10_4	Q2352	109.76	22.62	30899	453.20	0.83	27.71	650.10	17.96	19.60	199.70
Xs10_1	X80C2	125.20	29.05	29609	476.50	1.44	31.97	599.30	14.69	19.60	199.70
Xs10_2	X80C2	119.17	31.65	30960	486.50	1.00	30.20	609.50	17.81	19.60	199.70
Xs10_3	X80C2	134.37	27.48	29301	513.20	0.79	31.18	631.50	20.19	19.60	199.70
Xs10_4	X80C2	130.22	29.28	29595	483.20	1.28	29.34	608.10	15.41	19.60	199.70
Xs2_1	X80Cs2	120.84	27.40	29283	489.50	0.89	26.50	618.70	14.99	19.60	199.70
Xs2_2	X80Cs2	140.96	50.05	31209	508.50	0.84	28.28	596.30	16.54	19.60	199.70
Xs2_3	X80Cs2	110.19	30.01	30446	486.10	1.00	28.51	602.30	17.05	19.60	199.70
Ps10_1	PE	97.01	28.70	30980	414.00	0.43	27.92	622.40	19.98	11.50	159.20
Ps10_2	PE	91.73	22.33	29190	400.10	0.56	26.13	623.50	18.38	11.50	159.20
Ps10_3	PE	115.03	30.85	30280	401.30	0.61	26.91	609.50	20.32	11.50	159.20
Ps10_4	PE	83.50	32.55	30987	399.10	0.56	29.51	622.30	19.56	11.50	159.20
Ps10_5	PE	115.04	28.98	30404	401.50	0.56	27.01	646.40	18.24	11.50	159.20
Ps2_1	PEs	114.92	27.92	29930	392.40	0.48	27.01	631.50	22.58	11.50	159.20
Ps2_2	PEs	75.35	25.17	30215	383.40	0.44	27.89	641.30	19.05	11.50	159.20
Ps2_3	PEs	124.03	29.44	29504	398.50	0.50	27.50	625.30	16.53	11.50	159.20
Ps2_4	PEs	91.45	27.58	29977	390.40	0.50	28.71	669.10	17.97	11.50	159.20
Ps2_5	PEs	127.69	30.44	30582	405.30	0.61	27.07	609.30	21.20	11.50	159.20
Qs10_5	Q2353	139.23	32.60	30789	498.20	0.62	27.41	626.50	20.03	11.50	159.20
Qs10_6	Q2353	128.11	35.19	30974	422.50	0.72	28.68	609.60	18.95	11.50	159.20

Qs10_7	Q2353	145.31	28.89	29798	515.40	0.67	26.74	637.90	16.44	11.50	159.20
Qs2_5	Q235s3	198.15	34.52	31275	480.60	0.46	28.83	634.40	39.26	11.50	159.20
Qs2_6	Q235s3	233.44	39.73	31929	499.50	0.56	29.12	637.60	36.43	11.50	159.20
Qs2_7	Q235s3	180.02	34.30	31190	476.40	0.57	29.43	640.60	37.56	11.50	159.20
Xs2_5	X80Cs3	221.97	35.85	30501	512.60	0.60	30.04	664.50	16.56	11.50	159.20
Xs2_6	X80Cs3	189.62	31.87	29191	499.30	0.51	29.58	648.30	19.12	11.50	159.20
Xs2_7	X80Cs3	237.30	32.37	31310	510.30	0.67	30.63	631.20	18.55	11.50	159.20
Xs10_5	X80C3	149.30	25.74	30443	454.90	0.68	26.13	635.10	15.75	11.50	159.20
Xs10_6	X80C3	127.53	28.94	29141	501.60	0.67	27.47	643.10	17.55	11.50	159.20
Xs10_7	X80C3	137.17	26.59	31156	466.40	0.61	26.78	625.30	18.40	11.50	159.20

**sample ID**	**VWC （%）**	**pH**	**TN (g/kg)**	**TC (g/kg)**	**Cd (mg/kg)**	**weight_loss (mg)**	**corrosion rate (g mm^-2 month^-1)**	**redox (mv)**	**Amo-N (mg/kg)**	**NO2-N (mg/kg)**
S15	12.60	8.19	0.06	1.43	0.127935	N/A	N/A	N/A	8.0373	0.0567
S16	12.60	8.20	0.05	1.68	0.122101	N/A	N/A	N/A	8.0284	0.0582
S17	12.60	8.24	0.05	1.66	0.130075	N/A	N/A	N/A	7.6406	0.0825
S18	12.60	8.23	0.06	1.57	0.125953	N/A	N/A	N/A	7.6565	0.0768
S19	12.60	8.18	0.05	1.28	0.122287	N/A	N/A	N/A	7.6545	0.0804
S1	12.60	8.21	0.06	1.68	0.134161	N/A	N/A	N/A	7.4764	0.0435
S2	12.60	8.30	0.05	1.39	0.133597	N/A	N/A	N/A	7.4669	0.0447
S3	12.60	8.27	0.05	1.78	0.135802	N/A	N/A	N/A	7.4551	0.0453
S4	12.60	8.28	0.05	1.67	0.128604	N/A	N/A	N/A	7.4576	0.0429
S5	12.60	8.26	0.05	1.54	0.147302	N/A	N/A	N/A	7.4514	0.0438
S6	12.60	8.21	0.05	1.63	0.134773	N/A	N/A	N/A	7.1339	0.042
S7	12.60	8.14	0.05	1.48	0.129967	N/A	N/A	N/A	7.1281	0.03

S8	12.60	8.17	0.05	1.46	0.132853	N/A	N/A	N/A	7.1555	0.033
S9	12.60	8.22	0.05	1.54	0.118834	N/A	N/A	N/A	7.1428	0.036
S10	12.60	8.17	0.05	1.50	0.130099	N/A	N/A	N/A	7.1345	0.0537
S11	12.60	8.14	0.05	1.54	0.125227	N/A	N/A	N/A	7.1281	0.0546
S12	12.60	8.20	0.05	1.46	0.121802	N/A	N/A	N/A	7.1157	0.0543
S13	12.60	8.21	0.05	1.62	0.122377	N/A	N/A	N/A	7.1126	0.054
S14	12.60	8.18	0.05	1.63	0.125954	N/A	N/A	N/A	7.1251	0.0525
Qs2_1	26.00	8.39	0.05	1.51	0.125318	1.3765	0.2753	98.4	7.2676	0.0093
Qs2_2	26.00	8.37	0.05	1.50	0.13643	1.3935	0.2787	98	7.2791	0.0573
Qs2_3	26.00	8.40	0.05	1.53	0.127093	1.4	0.28	102.8	7.2704	0.0579
Qs2_4	26.00	8.39	0.05	1.51	0.129174	1.2705	0.2541	110	7.2776	0.0561
Qs10_1	26.00	8.51	0.05	1.56	0.122855	N/A	N/A	N/A	8.2743	0.0543
Qs10_2	26.00	8.48	0.05	1.57	0.125318	N/A	N/A	N/A	8.2621	0.0102
Qs10_3	26.00	8.47	0.05	1.58	0.132485	N/A	N/A	N/A	8.2678	0.0123
Qs10_4	26.00	8.52	0.05	1.56	0.106249	N/A	N/A	N/A	8.2714	0.0087
Xs10_1	26.00	8.38	0.05	1.61	0.121433	N/A	N/A	N/A	7.7058	0.0255
Xs10_2	26.00	8.42	0.05	1.57	0.132713	N/A	N/A	N/A	7.7172	0.0267
Xs10_3	26.00	8.40	0.05	1.61	0.12699	N/A	N/A	N/A	7.6789	0.0273
Xs10_4	26.00	8.38	0.05	1.57	0.125327	N/A	N/A	N/A	7.6801	0.036
Xs2_1	26.00	8.47	0.05	1.52	0.132713	1.7075	0.3415	92.24	7.7483	0.0663
Xs2_2	26.00	8.48	0.05	1.58	0.13412	1.523	0.3046	102.32	7.7592	0.0627
Xs2_3	26.00	8.47	0.05	1.56	0.140759	1.654	0.3308	92.1	7.7655	0.0663
Ps10_1	19.00	8.46	0.04	1.34	0.108071	N/A	N/A	N/A	6.8756	0.0339
Ps10_2	19.00	8.46	0.04	1.37	0.113769	N/A	N/A	N/A	6.8971	0.0288
Ps10_3	19.00	8.53	0.04	1.39	0.116913	N/A	N/A	N/A	6.8759	0.0345
Ps10_4	19.00	8.46	0.04	1.32	0.108109	N/A	N/A	N/A	6.8821	0.0288

Ps10_5	19.00	8.50	0.04	1.13	0.099625	N/A	N/A	N/A	6.8944	0.0327
Ps2_1	19.00	8.51	0.05	1.25	0.108869	0	0	0	6.8837	0.0333
Ps2_2	19.00	8.44	0.04	1.30	0.083013	0	0	0	6.8782	0.0339
Ps2_3	19.00	8.45	0.04	1.38	0.121969	0	0	0	6.8811	0.0327
Ps2_4	19.00	8.43	0.04	1.30	0.119432	0	0	0	6.8789	0.0324
Ps2_5	19.00	8.47	0.04	1.34	0.108081	0	0	0	6.8944	0.0366
Qs10_5	19.00	8.64	0.05	1.56	0.196759	N/A	N/A	N/A	7.5189	0.033
Qs10_6	19.00	8.57	0.04	1.52	0.120619	N/A	N/A	N/A	7.5263	0.0312
Qs10_7	19.00	8.65	0.05	1.50	0.111328	N/A	N/A	N/A	7.5303	0.0342
Qs2_5	19.00	8.54	0.04	1.47	0.539169	1.53	0.153	41.5	7.3291	0.0417
Qs2_6	19.00	8.50	0.04	1.62	0.478044	1.6765	0.16765	-0.7	7.3376	0.0402
Qs2_7	19.00	8.51	0.05	1.56	0.522778	2.2655	0.22655	20.4	7.3173	0.042
Xs2_5	19.00	8.57	0.04	2.16	0.116125	1.6945	0.16945	-4.9	7.3802	0
Xs2_6	19.00	8.53	0.05	2.14	0.160169	1.8215	0.18215	0.2	7.4014	0.0027
Xs2_7	19.00	8.50	0.04	1.97	0.130631	1.7265	0.17265	-6.2	7.4047	0.0033
Xs10_5	19.00	8.49	0.04	1.42	0.115941	N/A	N/A	N/A	6.8222	0.0264
Xs10_6	19.00	8.48	0.04	1.47	0.096043	N/A	N/A	N/A	6.8132	0.0273
Xs10_7	19.00	8.56	0.04	1.46	0.105673	N/A	N/A	N/A	6.8337	0.0264

VWC: water content, TN: Total Nitrogen, TC: Total Carbon, Amo-N: Ammonium-N, NO_2-N: Nitrite-N, N/A: No data.

Supplementary Table 2 Pairwise adonis test among among groups Q235 and Polymer at distance 2 cm and 10 cm of 5 and 10 months

number	pairs	F.Model	R2	p.value	p.adjusted
1	Q2352 vs Q235s2	3.094612	0.340269	0.022	0.038182
2	Q2352 vs Ps	11.15178	0.614363	0.005	0.03
3	Q2352 vs P	12.81767	0.64678	0.006	0.03
4	Q2352 vs Q2353	3.646507	0.421732	0.057	0.065769
5	Q2352 vs Q235s3	36.60691	0.879828	0.028	0.038182
6	Q235s2 vs Ps	11.89245	0.629482	0.004	0.03
7	Q235s2 vs P	12.6996	0.644663	0.011	0.038182
8	Q235s2 vs Q2353	3.015027	0.376172	0.032	0.04
9	Q235s2 vs Q235s3	15.8997	0.760762	0.027	0.038182
10	Ps vs P	0.69467	0.079896	0.594	0.594
11	Ps vs Q2353	6.27845	0.511339	0.027	0.038182
12	Ps vs Q235s3	35.83983	0.856596	0.02	0.038182
13	P vs Q2353	7.249491	0.547152	0.02	0.038182
14	P vs Q235s3	42.33947	0.875878	0.017	0.038182
15	Q2353 vs Q235s3	16.99844	0.80951	0.1	0.107143

Supplementary Table 3 Pairwise adonis test among among groups X80Cu and Polymer at distance 2 cm and 10 cm of 5 and 10 months

number	pairs	F.Model	R2	p.value	p.adjusted
1	X80Cu2 vs X80Cus2	2.706618814	0.351207044	0.032	0.053333333
2	X80Cu2 vs Ps	12.95209771	0.649159697	0.014	0.045
3	X80Cu2 vs P	15.12336181	0.683592392	0.012	0.045
4	X80Cu2 vs X80Cu3	5.787431916	0.536497654	0.055	0.075
5	X80Cu2 vs X80Cus3	13.62241181	0.731506313	0.024	0.045
6	X80Cus2 vs Ps	12.71431875	0.679389879	0.023	0.045
7	X80Cus2 vs P	13.43152616	0.69122343	0.021	0.045
8	X80Cus2 vs X80Cu3	5.579619109	0.582446864	0.1	0.107142857
9	X80Cus2 vs X80Cus3	12.40891191	0.756230028	0.1	0.107142857
10	Ps vs P	0.694670484	0.079896126	0.601	0.601
11	Ps vs X80Cu3	4.863759712	0.447705016	0.037	0.0555
12	Ps vs X80Cus3	22.6180577	0.790342166	0.016	0.045
13	P vs X80Cu3	7.055932022	0.540438784	0.023	0.045
14	P vs X80Cus3	27.4556934	0.820658328	0.01	0.045
15	X80Cu3 vs X80Cus3	15.00539302	0.789533424	0.1	0.107142857

Supplementary Table 4 Pairwise adonis test among groups at 2 cm and 10 cm from all materials of 10 months

number	pairs	F.Model	R2	p.value	p.adjusted
1	Ps vs P	0.694670484	0.079896126	0.611	0.611
2	Ps vs Q2353	6.278449672	0.511338959	0.03	0.06375
3	Ps vs Q235s3	35.83982567	0.856595961	0.013	0.05625
4	Ps vs X80Cu3	4.863759712	0.447705016	0.034	0.06375
5	Ps vs X80Cus3	22.6180577	0.790342166	0.026	0.06375
6	P vs Q2353	7.249491131	0.54715242	0.015	0.05625
7	P vs Q235s3	42.33947439	0.875877839	0.012	0.05625
8	P vs X80Cu3	7.055932022	0.540438784	0.013	0.05625
9	P vs X80Cus3	27.4556934	0.820658328	0.02	0.06
10	Q2353 vs Q235s3	16.99844299	0.809509686	0.1	0.115384615
11	Q2353 vs X80Cu3	1.863590371	0.317824107	0.3	0.321428571
12	Q2353 vs X80Cus3	7.286287692	0.645587627	0.1	0.115384615
13	Q235s3 vs X80Cu3	28.93029635	0.878531309	0.1	0.115384615
14	Q235s3 vs X80Cus3	15.81640611	0.798147052	0.1	0.115384615
15	X80Cu3 vs X80Cus3	15.00539302	0.789533424	0.1	0.115384615

Supplementary Table 5 Pairwise Adonis test of NMDS data of 5 months

number	pairs	F.Model	R2	p.value	p.adjusted
1	Q2352 vs Q235s2	3.730400856	0.383375866	0.03	0.05
2	Q2352 vs X80Cu2	0.884720645	0.128504945	0.82	0.82
3	Q2352 vs X80Cus2	3.732269827	0.427411189	0.03	0.05
4	Q235s2 vs X80Cu2	4.580184263	0.432902126	0.037	0.05
5	Q235s2 vs X80Cus2	1.042033575	0.172464049	0.366	0.4392
6	X80Cu2 vs X80Cus2	4.648630919	0.481791765	0.032	0.05

Supplementary Table 6 Pairwise Adonis test of NMDS data of 10 months

number	pairs	F.Model	R2	p.value	p.adjusted
1	Ps vs P	1.049581732	0.115981242	0.316	0.430909091
2	Ps vs Q2353	1.197030699	0.166322856	0.218	0.327
3	Ps vs Q235s3	1.635489415	0.214195754	0.07	0.327
4	Ps vs X80Cu3	1.782876816	0.229076839	0.056	0.327
5	Ps vs X80Cus3	1.285499731	0.176446336	0.183	0.327
6	P vs Q2353	1.002070836	0.14311064	0.362	0.4525
7	P vs Q235s3	1.663978894	0.217116842	0.094	0.327
8	P vs X80Cu3	2.326831672	0.279437818	0.073	0.327
9	P vs X80Cus3	1.402559932	0.189469581	0.177	0.327
10	Q2353 vs Q235s3	1.177798992	0.227470976	0.2	0.327
11	Q2353 vs X80Cu3	1.267259157	0.240591761	0.2	0.327
12	Q2353 vs X80Cus3	0.854234573	0.175977193	0.7	0.7
13	Q235s3 vs X80Cu3	1.503737622	0.273221168	0.2	0.327
14	Q235s3 vs X80Cus3	0.891455659	0.182247519	0.6	0.642857143
15	X80Cu3 vs X80Cus3	0.940422639	0.19035267	0.5	0.576923077

Supplementary Table 7 Top 50 OTUs in soil samples at 2 cm distance from Q235 and X80Cu coupon surface

OTUs	P1	Q2351	X80Cu1	Q235s2	Q2352	X80Cus2	X80Cu2	PE3	PEs3	Q235s3	Q2353	X80Cus3	X80Cu3
p__Proteobacteria;c__Betaproteobacteria;o__Gallionellales;f__Gallionellaceae;g__Gallionella	0.2	0.1	0.1	1437.2	15.7	591.6	26.4	2.3	0.0	3126.6	26.2	571.2	643.2
p__Proteobacteria;c__Betaproteobacteria;o__Burkholderiales;f__Comamonadaceae;g__Hydrogenophaga	3.0	4.5	1.6	1222.2	52.4	2064.0	165.3	0.2	8.6	276.7	1.7	9.6	233.1
p__Acidobacteria;c__Acidobacteria-6;o__iii1-15;f__;g__	847.9	767.8	747.1	180.7	457.6	189.5	522.3	1377.5	1420.0	768.9	1385.4	1319.2	1145.7
p__Nitrospirae;c__Nitrospira;o__Nitrospirales;f__Nitrospiraceae;g__Nitrospira	214.9	217.1	182.6	160.2	73.2	121.8	92.7	196.3	266.0	617.1	214.5	495.0	411.2
p__Proteobacteria;c__Deltaproteobacteria;o__Syntrophobacterales;f__Syntrophobacteraceae;g__	813.0	820.0	858.8	67.6	255.5	54.0	343.2	862.1	759.9	383.8	822.5	706.7	740.8
p__Proteobacteria;c__Gammaproteobacteria;o__Xanthomonadales;f__Xanthomonadaceae;g__Lysobacter	12.1	15.8	16.4	285.0	15.0	490.2	22.9	52.7	84.7	283.2	39.7	55.9	92.9
p__Bacteroidetes;c__Cytophagia;o__Cytophagales;f__Cytophagaceae;g__	41.9	47.4	39.3	284.7	122.3	190.2	155.8	97.5	176.5	388.5	214.0	399.6	230.4
p__Nitrospirae;c__Nitrospira;o__Nitrospirales;f__0319-6A21;g__	339.2	295.4	310.6	85.0	122.6	101.9	138.8	601.7	373.0	456.5	648.2	409.6	473.7
p__Proteobacteria;c__Gammaproteobacteria;o__Xanthomonadales;f__Xanthomonadaceae;g__Thermomonas	18.8	23.3	14.0	343.2	22.2	365.2	36.1	38.9	124.2	267.7	38.9	58.0	54.4
p__Firmicutes;c__Bacilli;o__Lactobacillales;f__Streptococcaceae;g__Lactococcus	2109.2	2166.1	2053.9	132.7	0.8	53.4	0.3	404.1	278.4	397.5	722.0	747.8	501.2

p__Acidobacteria;c__DA052;o__Ellin6513;f__;g__	485.6	480.2	597.1	39.5	94.0	28.7	106.2	1107.6	705.7	482.7	648.7	307.9	502.5
p__Proteobacteria;c__Betaproteobacteria;o__SBla14;f__;g__	0.0	0.0	0.0	454.7	23.5	321.9	80.8	7.0	0.6	68.2	15.9	2.4	14.7
p__Proteobacteria;c__Betaproteobacteria;o__;f__;g__	363.1	335.5	383.3	62.2	133.2	43.9	127.9	758.4	737.8	431.4	637.1	424.7	398.6
p__Proteobacteria;c__Betaproteobacteria;o__MND1;f__;g__	121.2	127.0	109.5	55.2	92.0	39.5	81.1	231.4	253.3	350.8	509.0	354.7	305.5
p__Proteobacteria;c__Alphaproteobacteria;o__Rhodospirillales;f__Rhodospirillaceae;g__	116.4	105.9	107.5	131.2	55.1	99.1	58.7	169.7	197.0	313.0	190.7	220.9	175.6
p__Actinobacteria;c__Thermoleophilia;o__Gaiellales;f__Gaiellaceae;g__	216.5	163.4	196.8	125.0	116.9	94.8	103.7	220.3	163.8	219.8	323.0	282.2	274.1
p__Chloroflexi;c__Anaerolineae;o__envOPS12;f__;g__	393.8	342.1	354.5	65.0	252.2	40.5	284.8	348.1	358.8	268.9	457.9	356.2	322.1
p__Crenarchaeota;c__Thaumarchaeota;o__Nitrososphaerales;f__Nitrososphaeraceae;g__Nitrososphaera	229.0	142.0	131.9	28.1	371.8	7.1	285.7	146.0	195.0	144.2	323.1	663.0	525.7
p__Gemmatimonadetes;c__Gemm-1;o__;f__;g__	179.3	134.9	143.3	27.6	61.3	29.3	53.0	218.9	167.0	249.8	377.3	263.9	315.1
p__Proteobacteria;c__Betaproteobacteria;o__Burkholderiales;f__Comamonadaceae;g__Limnohabitans	11.7	14.0	17.3	168.3	35.3	187.3	37.3	10.8	21.0	161.9	22.2	27.1	42.2
p__Proteobacteria;c__Betaproteobacteria;o__Burkholderiales;f__Comamonadaceae;Other	21.4	24.2	21.9	160.2	51.7	180.4	41.7	27.7	34.1	160.3	26.4	46.7	52.5
k__Archaea;p__Crenarchaeota;c__Thaumarchaeota;o__Cenarchaeales;f__Cenarchaeaceae;g__	24.7	24.2	47.9	46.3	31.6	8.4	23.5	56.2	104.3	289.3	168.1	176.9	244.5
p__GAL15;c__;o__;f__;g__	479.3	272.4	285.5	32.9	96.7	53.3	134.7	398.9	225.2	245.7	402.0	275.6	208.0

p__Nitrospirae;c__Nitrospira;o__Nitrospirales;f__Nitrospiraceae;g__	339.7	300.1	350.0	24.3	58.4	22.9	85.6	470.0	345.3	201.3	364.9	258.4	247.5
p__Acidobacteria;c__DA052;o__E29;f__;g__	247.8	219.4	303.3	15.9	33.6	18.2	47.0	580.6	376.7	232.1	412.5	187.3	228.8
p__Acidobacteria;c__Acidobacteria-6;o__iii1-15;f__mb2424;g__	143.5	128.2	109.4	56.9	72.8	49.6	84.7	123.8	186.3	133.5	195.3	208.1	209.8
p__Acidobacteria;c__Acidobacteria-6;o__CCU21;f__;g__	129.6	106.4	107.6	66.5	53.4	73.2	54.0	189.5	106.9	158.7	260.2	126.1	146.0
p__Acidobacteria;c__S035;o__;f__;g__	299.2	295.4	262.9	35.2	155.8	24.6	182.5	318.1	315.4	131.4	278.7	295.8	262.4
p__Chloroflexi;c__Anaerolineae;o__SB-34;f__;g__	382.0	287.5	364.6	44.5	77.6	31.0	123.3	363.6	242.1	182.4	385.7	178.8	180.1
p__Proteobacteria;c__Betaproteobacteria;o__Nitrosomonadales;f__Nitrosomonadaceae;g__	0.0	0.1	0.4	81.0	0.5	73.2	0.5	0.0	1.2	215.2	4.4	52.5	46.9
p__Acidobacteria;c__[Chloracidobacteria];o__RB41;f__;g__	243.0	188.8	159.9	15.9	163.8	12.8	159.9	169.3	254.5	89.8	155.2	319.1	314.3
p__Planctomycetes;c__Planctomycetia;o__Pirellulales;f__Pirellulaceae;g__	229.8	204.2	185.1	52.9	131.1	28.3	152.6	139.6	154.9	106.1	238.3	245.1	209.4
p__NC10;c__wb1-A12;o__;f__;g__	295.0	258.9	256.8	15.2	48.5	19.6	62.5	561.1	388.8	176.3	309.9	181.9	193.8
p__Proteobacteria;c__Betaproteobacteria;o__Rhodocyclales;f__Rhodocyclaceae;g__Dechloromonas	0.2	0.3	0.4	53.8	3.4	47.9	3.1	1.0	0.0	256.8	2.8	2.7	15.7
p__Proteobacteria;c__Gammaproteobacteria;o__Xanthomonadales;f__Sinobacteraceae;g__	56.6	59.2	44.9	38.5	64.5	34.4	63.4	119.0	155.0	141.3	196.5	233.4	147.1
p__Acidobacteria;c__Solibacteres;o__Solibacterales;f__PAUC26f;g__	137.5	153.5	174.6	13.4	54.0	8.4	57.4	467.2	274.0	134.8	265.8	162.0	191.2

p__Firmicutes;c__Bacilli;o__Bacillales;f__Bacillaceae;g__Bacillus	6667.9	6955.6	6917.6	42.3	37.5	20.6	44.5	102.7	72.5	104.5	182.7	227.8	161.3
p__Bacteroidetes;c__Sphingobacteriia;o__Sphingobacteriales;f__;g__	11.9	17.0	10.2	57.2	115.3	26.3	163.4	52.3	122.8	103.8	113.5	241.7	129.0
p__Proteobacteria;c__Betaproteobacteria;o__Rhodocyclales;f__Rhodocyclaceae;g__Dok59	0.0	0.0	0.1	35.9	0.0	124.7	1.3	0.0	1.0	66.1	9.9	140.8	90.9
p__NC10;c__12-24;o__JH-WHS47;f__;g__	173.0	141.8	167.3	23.8	30.8	20.9	41.1	306.9	228.2	135.8	212.2	106.9	135.1
k__Archaea;p__Crenarchaeota;c__Thaumarchaeota;o__Cenarchaeales;f__SAGMA-X;g__	129.1	129.9	204.9	5.6	54.3	2.4	53.2	284.2	182.0	82.6	312.0	196.9	221.7
p__Chloroflexi;c__Anaerolineae;o__H39;f__;g__	161.3	125.8	170.9	15.4	44.5	10.8	42.7	210.7	144.9	122.0	222.5	125.8	158.4
p__Chloroflexi;c__S085;o__;f__;g__	165.0	133.6	155.0	21.0	39.4	23.3	40.2	144.9	101.3	116.9	173.8	117.6	119.7
p__WS3;c__PRR-12;o__Sediment-1;f__;g__	95.3	84.7	81.5	16.4	38.3	15.9	54.2	190.8	121.9	95.7	192.1	106.5	146.0
p__Proteobacteria;c__Alphaproteobacteria;o__Rhizobiales;f__Hyphomicrobiaceae;g__Rhodoplanes	28.9	41.1	30.6	67.7	27.1	41.8	24.7	32.2	38.4	72.9	75.8	77.7	58.7
p__Actinobacteria;c__MB-A2-108;o__0319-7L14;f__;g__	60.8	51.5	36.4	45.8	40.4	46.2	40.2	59.2	38.1	74.2	106.5	107.6	77.3
p__Proteobacteria;c__Betaproteobacteria;o__IS-44;f__;g__	57.5	59.6	74.0	39.4	19.3	27.0	19.6	113.8	170.2	103.1	93.2	58.4	75.4
p__Proteobacteria;c__Betaproteobacteria;o__Burkholderiales;f__Comamonadaceae;g__Methylibium	8.7	8.8	7.8	73.5	33.1	47.2	22.1	12.2	19.1	82.8	25.7	22.0	28.3
p__Proteobacteria;c__Betaproteobacteria;o__Ellin6067;f__;g__	2.4	2.9	3.2	58.4	9.8	53.9	6.2	12.5	27.8	102.5	50.0	30.6	21.8

Supplementary Table 8 Results of random forest modeling using weight loss ratio data

OTUs	% IncMSE	% IncMSE.pval	IncNode Purity	IncNodePurity.pval
k__Bacteria;p__Proteobacteria;c__Betaproteobacteria;o__Gallionellales;f__Gallionellaceae;g__Gallionella	2.91	0.02	1.81	0.02
k__Bacteria;p__Bacteroidetes;c__[Saprospirae];o__[Saprospirales];f__Chitinophagaceae;g__Lacibacter	2.83	0.02	3.09	0.02
k__Bacteria;p__Proteobacteria;c__Betaproteobacteria;o__Rhodocyclales;f__Rhodocyclaceae;g__Dechloromonas	2.47	0.02	1.42	0.02
k__Bacteria;p__Actinobacteria;c__Acidimicrobiia;o__Acidimicrobiales;f__Iamiaceae;g__Iamia	1.94	0.10	0.65	0.22
k__Bacteria;p__Actinobacteria;c__Thermoleophilia;o__Solirubrobacterales;f__;g__	1.84	0.02	0.56	0.20
k__Bacteria;p__Proteobacteria;c__Betaproteobacteria;o__Nitrosomonadales;f__Nitrosomonadaceae;g__	1.67	0.06	0.55	0.08
k__Bacteria;p__Proteobacteria;c__Betaproteobacteria;o__Burkholderiales;f__Comamonadaceae;g__Hydrogenophaga	1.66	0.06	0.39	0.27
k__Bacteria;p__Actinobacteria;c__Thermoleophilia;o__Gaiellales;f__;g__	1.63	0.06	0.67	0.08
k__Archaea;p__Euryarchaeota;c__DSEG;o__HydGC-84-221A;f__;g__	1.57	0.06	0.08	0.43
k__Bacteria;p__Firmicutes;c__Bacilli;o__Lactobacillales;f__Streptococcaceae;g__Lactococcus	1.36	0.16	0.19	0.37
k__Bacteria;p__Chloroflexi;c__TK10;o__B07_WMSP1;f__;g__	1.32	0.10	0.13	0.47
k__Bacteria;p__Firmicutes;c__Clostridia;o__Clostridiales;f__Symbiobacteriaceae;g__Symbiobacterium	1.31	0.08	0.00	0.82
k__Bacteria;p__Firmicutes;c__Bacilli;o__Lactobacillales;f__Streptococcaceae;g__Streptococcus	1.28	0.18	0.04	0.88
k__Bacteria;p__Proteobacteria;c__Deltaproteobacteria;o__Myxococcales;f__Myxococcaceae;g__Corallococcus	1.28	0.12	0.01	0.92
k__Bacteria;p__Chloroflexi;c__TK10;o__AKYG885;f__Dolo_23;g__	1.19	0.18	0.34	0.18
k__Bacteria;p__Proteobacteria;c__Betaproteobacteria;o__Rhodocyclales;f__Rhodocyclaceae;g__Azospira	1.03	0.27	0.01	0.80
k__Bacteria;p__Proteobacteria;c__Gammaproteobacteria;o__Xanthomonadales;f__Xanthomonadaceae;g__Thermomonas	1.02	0.14	0.06	0.86
k__Bacteria;p__Cyanobacteria;c__ML635J-21;o__;f__;g__	1.01	0.12	0.00	0.88
k__Bacteria;p__BHI80-139;c__;o__;f__;g__	1.01	0.25	0.02	0.88
k__Bacteria;p__Proteobacteria;c__Alphaproteobacteria;o__Caulobacterales;f__Caulobacteraceae;g__Mycoplana	1.01	0.27	0.01	0.67
k__Bacteria;p__Proteobacteria;c__Betaproteobacteria;o__SBla14;f__;g__	1.01	0.35	0.00	0.88
k__Bacteria;p__Proteobacteria;c__Gammaproteobacteria;o__Xanthomonadales;f__Xanthomonadaceae;g__Lysobacter	1.01	0.27	0.03	0.76

k__Bacteria;p__Nitrospirae;c__Nitrospira;o__Nitrospirales;f__Nitrospiraceae;g__Nitrospira	1.01	0.20	0.04	0.90
k__Bacteria;p__Bacteroidetes;c__Cytophagia;o__Cytophagales;f__Cyclobacteriaceae;g__	1.01	0.24	0.01	0.76
k__Bacteria;p__Proteobacteria;c__Alphaproteobacteria;o__Caulobacterales;f__Caulobacteraceae;g__	0.97	0.37	0.02	0.73
k__Bacteria;p__Proteobacteria;c__Betaproteobacteria;o__Burkholderiales;f__Comamonadaceae;Other	0.96	0.25	0.23	0.27
k__Bacteria;p__Proteobacteria;c__Gammaproteobacteria;o__Legionellales;f__Legionellaceae;Other	0.80	0.41	0.01	0.78
k__Bacteria;p__Proteobacteria;c__Alphaproteobacteria;o__Rhizobiales;f__Bradyrhizobiaceae;g__Bradyrhizobium	0.70	0.37	0.50	0.24
k__Bacteria;p__Proteobacteria;c__Gammaproteobacteria;o__Xanthomonadales;f__Xanthomonadaceae;g__Arenimonas	0.68	0.35	0.03	0.90
k__Bacteria;p__Proteobacteria;c__Alphaproteobacteria;o__Rhodospirillales;f__Rhodospirillaceae;g__	0.52	0.31	0.09	0.78
k__Bacteria;p__Proteobacteria;c__Betaproteobacteria;o__Rhodocyclales;f__Rhodocyclaceae;g__Sulfuritalea	0.50	0.29	0.01	0.65
k__Bacteria;p__Proteobacteria;c__Deltaproteobacteria;o__DTB120;f__;g__	0.25	0.39	0.34	0.14
k__Bacteria;p__Chloroflexi;c__Ellin6529;o__;f__;g__	0.17	0.33	0.45	0.16
k__Bacteria;p__Proteobacteria;c__Betaproteobacteria;o__IS-44;f__;g__	0.14	0.43	0.73	0.20
k__Bacteria;p__Bacteroidetes;c__[Saprospirae];o__[Saprospirales];f__Chitinophagaceae;g__Sediminibacterium	0.03	0.27	0.01	0.73
k__Bacteria;p__Actinobacteria;c__Actinobacteria;o__Micrococcales;f__;g__	0.00	0.49	0.00	0.96
k__Bacteria;p__Firmicutes;c__Bacilli;o__Lactobacillales;f__Leuconostocaceae;g__Leuconostoc	0.00	0.51	0.04	0.78
k__Bacteria;p__Proteobacteria;c__Alphaproteobacteria;o__Rhizobiales;f__Hyphomicrobiaceae;g__	0.00	0.37	0.02	0.90
k__Bacteria;p__Proteobacteria;c__Alphaproteobacteria;o__Rhizobiales;f__Hyphomicrobiaceae;g__Rhodoplanes	0.00	0.41	0.00	1.00
k__Bacteria;p__Proteobacteria;c__Betaproteobacteria;o__Ellin6067;f__;g__	0.00	0.61	0.00	0.94
k__Bacteria;p__Proteobacteria;c__Deltaproteobacteria;o__Desulfobacterales;f__Desulfobulbaceae;g__	0.00	0.57	0.00	1.00
k__Bacteria;p__Proteobacteria;c__Gammaproteobacteria;o__Xanthomonadales;f__Xanthomonadaceae;g__Dokdonella	0.00	0.61	0.00	1.00
k__Bacteria;p__Proteobacteria;c__Alphaproteobacteria;o__Rhizobiales;f__Xanthobacteraceae;g__Xanthobacter	0.00	1.00	0.02	0.31
k__Bacteria;p__Proteobacteria;c__Gammaproteobacteria;o__Xanthomonadales;f__Xanthomonadaceae;g__	-0.73	0.69	0.14	0.69
k__Bacteria;p__Actinobacteria;c__Actinobacteria;o__Actinomycetales;f__Micromonosporaceae;g__	-0.82	0.67	0.01	0.78
k__Bacteria;p__Proteobacteria;c__Betaproteobacteria;o__Burkholderiales;f__Comamonadaceae;g__Methylibium	-0.83	0.63	1.32	0.06
k__Bacteria;p__Proteobacteria;c__Alphaproteobacteria;o__Rhodospirillales;f__Rhodospirillaceae;g__Magnetospirillum	-0.89	0.59	0.01	0.57
k__Bacteria;p__Proteobacteria;c__Alphaproteobacteria;o__Rhizobiales;f__Hyphomicrobiaceae;g__Devosia	-0.93	0.71	0.15	0.45

k__Bacteria;p__Proteobacteria;c__Gammaproteobacteria;o__Legionellales;f__;g__	-0.94	0.69	0.12	0.76
k__Bacteria;p__Actinobacteria;c__Actinobacteria;o__Actinomycetales;f__Sporichthyaceae;g__	-1.01	0.88	0.00	0.92
k__Bacteria;p__Proteobacteria;c__Betaproteobacteria;o__Burkholderiales;f__Comamonadaceae;g__Limnohabitans	-1.01	0.82	0.29	0.24
k__Bacteria;p__Proteobacteria;c__Gammaproteobacteria;o__Xanthomonadales;f__Xanthomonadaceae;g__Luteimonas	-1.01	0.86	0.11	0.53
k__Bacteria;p__Proteobacteria;c__Betaproteobacteria;o__Burkholderiales;f__Comamonadaceae;g__Leptothrix	-1.01	0.71	0.19	0.31
k__Bacteria;p__Chloroflexi;c__Thermomicrobia;o__AKYG1722;f__;g__	-1.01	0.76	0.08	0.69
k__Bacteria;p__Verrucomicrobia;c__Opitutae;o__Opitutales;f__Opitutaceae;g__Opitutus	-1.01	0.73	0.02	0.84
k__Bacteria;p__Gemmatimonadetes;c__Gemmatimonadetes;o__;f__;g__	-1.01	0.73	0.04	0.88
k__Bacteria;p__Actinobacteria;c__Thermoleophilia;o__Solirubrobacterales;f__Solirubrobacteraceae;g__	-1.02	0.69	0.00	0.98
k__Bacteria;p__Proteobacteria;c__Betaproteobacteria;o__Methylophilales;f__;g__	-1.04	0.84	0.04	0.69
k__Bacteria;p__Bacteroidetes;c__Cytophagia;o__Cytophagales;f__Cytophagaceae;g__	-1.09	0.78	0.08	0.76
k__Bacteria;p__Proteobacteria;c__Gammaproteobacteria;o__Xanthomonadales;f__Sinobacteraceae;g__Nevskia	-1.11	0.96	0.00	0.69
k__Bacteria;p__Firmicutes;c__Clostridia;o__Clostridiales;f__Peptococcaceae;g__Desulfosporosinus	-1.16	0.92	0.07	0.43
k__Bacteria;p__Proteobacteria;c__Betaproteobacteria;o__Rhodocyclales;f__Rhodocyclaceae;g__Dok59	-1.34	0.92	0.18	0.33
k__Bacteria;p__Verrucomicrobia;c__Opitutae;o__Opitutales;f__Opitutaceae;g__	-1.43	0.94	0.00	0.96
k__Bacteria;p__Actinobacteria;c__Actinobacteria;o__Actinomycetales;f__Nocardioidaceae;g__Pimelobacter	-1.44	0.92	0.01	0.82
k__Bacteria;p__Proteobacteria;c__Alphaproteobacteria;o__Rhizobiales;f__Phyllobacteriaceae;g__Mesorhizobium	-1.57	0.98	0.09	0.76
k__Bacteria;p__Proteobacteria;c__Betaproteobacteria;o__Burkholderiales;f__Comamonadaceae;g__Variovorax	-1.65	0.92	0.21	0.63

Supplementary Table 9 Results of random forest modeling using Redox data

OTUs	%IncMSE	%IncMSE.pval	IncNodePurity	IncNodePurity.pval
k__Bacteria;p__Proteobacteria;c__Alphaproteobacteria;o__Caulobacterales;f__Caulobacteraceae;g__	3.22	0.02	4731.11	0.02
k__Bacteria;p__Proteobacteria;c__Betaproteobacteria;o__SBla14;f__;g__	2.88	0.02	3594.86	0.02
k__Bacteria;p__Proteobacteria;c__Betaproteobacteria;o__Burkholderiales;f__Comamonadaceae;g__Hydrogenophaga	2.59	0.02	3868.33	0.02
k__Bacteria;p__Bacteroidetes;c__Cytophagia;o__Cytophagales;f__Cyclobacteriaceae;g__	2.01	0.02	1280.44	0.02
k__Bacteria;p__Proteobacteria;c__Gammaproteobacteria;o__Legionellales;f__Legionellaceae;Other	1.91	0.02	2367.29	0.06
k__Bacteria;p__Bacteroidetes;c__[Saprospirae];o__[Saprospirales];f__Chitinophagaceae;g__Lacibacter	1.70	0.04	1367.25	0.06
k__Bacteria;p__Proteobacteria;c__Alphaproteobacteria;o__Rhizobiales;f__Hyphomicrobiaceae;g__Devosia	1.70	0.04	1641.88	0.02
k__Bacteria;p__Proteobacteria;c__Betaproteobacteria;o__Burkholderiales;f__Comamonadaceae;g__Leptothrix	1.58	0.08	42.07	0.82
k__Bacteria;p__Nitrospirae;c__Nitrospira;o__Nitrospirales;f__Nitrospiraceae;g__Nitrospira	1.49	0.06	956.77	0.22
k__Bacteria;p__Proteobacteria;c__Alphaproteobacteria;o__Rhodospirillales;f__Rhodospirillaceae;g__	1.40	0.12	1465.67	0.22
k__Bacteria;p__Proteobacteria;c__Betaproteobacteria;o__IS-44;f__;g__	1.35	0.08	2120.14	0.08
k__Bacteria;p__Proteobacteria;c__Betaproteobacteria;o__Rhodocyclales;f__Rhodocyclaceae;g__Dok59	1.33	0.10	4.28	0.78
k__Bacteria;p__Firmicutes;c__Bacilli;o__Lactobacillales;f__Streptococcaceae;g__Lactococcus	1.26	0.08	2349.66	0.06
k__Bacteria;p__Proteobacteria;c__Deltaproteobacteria;o__DTB120;f__;g__	1.19	0.20	2.39	0.86
k__Bacteria;p__Proteobacteria;c__Betaproteobacteria;o__Burkholderiales;f__Comamonadaceae;g__Methylibium	1.14	0.18	837.51	0.29
k__Bacteria;p__Actinobacteria;c__Thermoleophilia;o__Gaiellales;f__;g__	1.06	0.18	13.55	0.90
k__Bacteria;p__Gemmatimonadetes;c__Gemmatimonadetes;o__;f__;g__	1.04	0.16	1541.60	0.08
k__Bacteria;p__Actinobacteria;c__Thermoleophilia;o__Solirubrobacterales;f__;g__	1.02	0.14	17.96	0.92
k__Bacteria;p__Actinobacteria;c__Actinobacteria;o__Micrococcales;f__;g__	1.01	0.18	447.65	0.55
k__Bacteria;p__Proteobacteria;c__Alphaproteobacteria;o__Rhizobiales;f__Hyphomicrobiaceae;g__	1.01	0.10	2.07	0.98
k__Bacteria;p__Proteobacteria;c__Betaproteobacteria;o__Rhodocyclales;f__Rhodocyclaceae;g__Sulfuritalea	1.01	0.27	1.55	0.65

k__Bacteria;p__Proteobacteria;c__Gammaproteobacteria;o__Xanthomonadales;f__Sinobacteraceae;g__Nevskia	1.01	0.18	582.55	0.06
k__Bacteria;p__Firmicutes;c__Clostridia;o__Clostridiales;f__Symbiobacteriaceae;g__Symbiobacterium	1.00	0.37	323.48	0.25
k__Bacteria;p__Proteobacteria;c__Gammaproteobacteria;o__Xanthomonadales;f__Xanthomonadaceae;g__Lysobacter	1.00	0.16	404.56	0.53
k__Bacteria;p__Proteobacteria;c__Alphaproteobacteria;o__Rhizobiales;f__Hyphomicrobiaceae;g__Rhodoplanes	0.97	0.22	1.32	0.98
k__Bacteria;p__Proteobacteria;c__Betaproteobacteria;o__Burkholderiales;f__Comamonadaceae;Other	0.91	0.39	3.01	0.90
k__Bacteria;p__Proteobacteria;c__Betaproteobacteria;o__Rhodocyclales;f__Rhodocyclaceae;g__Dechloromonas	0.90	0.31	381.28	0.25
k__Bacteria;p__Chloroflexi;c__Ellin6529;o__;f__;g__	0.85	0.22	454.98	0.63
k__Bacteria;p__Proteobacteria;c__Alphaproteobacteria;o__Caulobacterales;f__Caulobacteraceae;g__Mycoplana	0.82	0.35	834.33	0.02
k__Bacteria;p__Firmicutes;c__Bacilli;o__Lactobacillales;f__Streptococcaceae;g__Streptococcus	0.34	0.37	1355.56	0.16
k__Bacteria;p__Proteobacteria;c__Betaproteobacteria;o__Nitrosomonadales;f__Nitrosomonadaceae;g__	0.28	0.33	25.53	0.94
k__Bacteria;p__Proteobacteria;c__Betaproteobacteria;o__Gallionellales;f__Gallionellaceae;g__Gallionella	0.12	0.53	26.16	0.73
k__Bacteria;p__Proteobacteria;c__Gammaproteobacteria;o__Legionellales;f__;g__	0.00	0.45	4.37	0.96
k__Bacteria;p__BHI80-139;c__;o__;f__;g__	0.00	0.43	1.59	0.98
k__Bacteria;p__Cyanobacteria;c__ML635J-21;o__;f__;g__	0.00	0.61	0.87	0.84
k__Bacteria;p__Proteobacteria;c__Deltaproteobacteria;o__Myxococcales;f__Myxococcaceae;g__Corallococcus	0.00	0.31	0.33	1.00
k__Bacteria;p__Proteobacteria;c__Gammaproteobacteria;o__Xanthomonadales;f__Xanthomonadaceae;g__Dokdonella	0.00	0.37	2.14	0.96
k__Bacteria;p__Actinobacteria;c__Actinobacteria;o__Actinomycetales;f__Micromonosporaceae;g__	0.00	0.47	0.01	0.96
k__Bacteria;p__Bacteroidetes;c__Cytophagia;o__Cytophagales;f__Cytophagaceae;g__	0.00	0.49	83.47	0.92
k__Bacteria;p__Chloroflexi;c__Thermomicrobia;o__AKYG1722;f__;g__	0.00	0.37	13.14	0.98
k__Bacteria;p__Proteobacteria;c__Alphaproteobacteria;o__Rhizobiales;f__Xanthobacteraceae;g__Xanthobacter	0.00	1.00	6.40	0.51
k__Bacteria;p__Verrucomicrobia;c__Opitutae;o__Opitutales;f__Opitutaceae;g__Opitutus	0.00	0.39	388.07	0.49
k__Archaea;p__Euryarchaeota;c__DSEG;o__HydGC-84-221A;f__;g__	0.00	0.57	0.00	0.90
k__Bacteria;p__Proteobacteria;c__Betaproteobacteria;o__Methylophilales;f__;g__	0.00	0.49	0.00	1.00
k__Bacteria;p__Bacteroidetes;c__[Saprospirae];o__[Saprospirales];f__Chitinophagaceae;g__Sediminibacterium	-0.09	0.71	434.65	0.14
k__Bacteria;p__Proteobacteria;c__Betaproteobacteria;o__Rhodocyclales;f__Rhodocyclaceae;g__Azospira	-0.17	0.45	29.58	0.80
k__Bacteria;p__Verrucomicrobia;c__Opitutae;o__Opitutales;f__Opitutaceae;g__	-0.19	0.55	44.14	0.98

k__Bacteria;p__Actinobacteria;c__Actinobacteria;o__Actinomycetales;f__Nocardioidaceae;g__Pimelobacter	-0.22	0.41	98.88	0.90
k__Bacteria;p__Proteobacteria;c__Deltaproteobacteria;o__Desulfobacterales;f__Desulfobulbaceae;g__	-0.64	0.61	0.27	0.92
k__Bacteria;p__Firmicutes;c__Clostridia;o__Clostridiales;f__Peptococcaceae;g__Desulfosporosinus	-0.77	0.67	29.47	0.84
k__Bacteria;p__Proteobacteria;c__Betaproteobacteria;o__Burkholderiales;f__Comamonadaceae;g__Limnohabitans	-0.80	0.63	44.69	0.80
k__Bacteria;p__Proteobacteria;c__Betaproteobacteria;o__Burkholderiales;f__Comamonadaceae;g__Variovorax	-0.82	0.55	90.30	0.92
k__Bacteria;p__Proteobacteria;c__Gammaproteobacteria;o__Xanthomonadales;f__Xanthomonadaceae;g__Luteimonas	-0.95	0.75	276.67	0.59
k__Bacteria;p__Chloroflexi;c__TK10;o__B07_WMSP1;f__;g__	-0.97	0.69	41.56	0.86
k__Bacteria;p__Proteobacteria;c__Alphaproteobacteria;o__Rhizobiales;f__Bradyrhizobiaceae;g__Bradyrhizobium	-0.98	0.82	13.84	0.90
k__Bacteria;p__Actinobacteria;c__Acidimicrobiia;o__Acidimicrobiales;f__Iamiaceae;g__Iamia	-1.00	0.71	30.08	0.96
k__Bacteria;p__Actinobacteria;c__Actinobacteria;o__Actinomycetales;f__Sporichthyaceae;g__	-1.01	0.76	0.00	1.00
k__Bacteria;p__Actinobacteria;c__Thermoleophilia;o__Solirubrobacterales;f__Solirubrobacteraceae;g__	-1.01	0.80	268.15	0.80
k__Bacteria;p__Chloroflexi;c__TK10;o__AKYG885;f__Dolo_23;g__	-1.01	0.73	0.00	1.00
k__Bacteria;p__Proteobacteria;c__Alphaproteobacteria;o__Rhodospirillales;f__Rhodospirillaceae;g__Magnetospirillum	-1.08	0.88	866.08	0.04
k__Bacteria;p__Firmicutes;c__Bacilli;o__Lactobacillales;f__Leuconostocaceae;g__Leuconostoc	-1.22	0.88	1107.03	0.27
k__Bacteria;p__Proteobacteria;c__Betaproteobacteria;o__Ellin6067;f__;g__	-1.24	0.92	0.94	0.96
k__Bacteria;p__Proteobacteria;c__Alphaproteobacteria;o__Rhizobiales;f__Phyllobacteriaceae;g__Mesorhizobium	-1.42	0.94	651.87	0.33
k__Bacteria;p__Proteobacteria;c__Gammaproteobacteria;o__Xanthomonadales;f__Xanthomonadaceae;g__Thermomonas	-1.62	0.98	201.20	0.71
k__Bacteria;p__Proteobacteria;c__Gammaproteobacteria;o__Xanthomonadales;f__Xanthomonadaceae;g__Arenimonas	-1.78	0.98	137.59	1.00
k__Bacteria;p__Proteobacteria;c__Gammaproteobacteria;o__Xanthomonadales;f__Xanthomonadaceae;g__	-1.98	1.00	140.04	0.76

Supplementary Table 10 nodes and edges information of 2 cm co-occurrence network

Id	name	modularity_class	degree	betweenness	closeness	eigenvector
0	k__Archaea;p__Crenarchaeota;c__Thaumarchaeota;o__AK31;f__;g__	2	133	1.00	0.0045	0.9676
1	k__Archaea;p__Crenarchaeota;c__Thaumarchaeota;o__Cenarchaeales;f__Cenarchaeaceae;g__	2	64	560.00	0.0043	0.3677
2	k__Archaea;p__Crenarchaeota;c__Thaumarchaeota;o__Cenarchaeales;f__Cenarchaeaceae;g__Nitrosopumilus	2	66	13.00	0.0041	0.3933
3	k__Archaea;p__Crenarchaeota;c__Thaumarchaeota;o__Cenarchaeales;f__SAGMA-X;g__	2	124	15.00	0.0043	0.8933
4	k__Archaea;p__Crenarchaeota;c__Thaumarchaeota;o__Nitrososphaerales;f__Nitrososphaeraceae;g__Candidatus Nitrososphaera	0	134	0.00	0.0045	0.9740
5	k__Archaea;p__Euryarchaeota;c__Thermoplasmata;o__E2;f__;g__	2	115	40.00	0.0047	0.7472
6	k__Archaea;p__Euryarchaeota;c__Thermoplasmata;o__E2;f__[Methanomassiliicoccaceae];g__	2	129	11.00	0.0044	0.9500
7	k__Archaea;p__[Parvarchaeota];c__[Parvarchaea];o__YLA114;f__;g__	2	135	1.00	0.0045	0.9950
8	k__Bacteria;Other;Other;Other;Other;Other	0	132	0.00	0.0045	0.9552
9	k__Bacteria;p__Acidobacteria;c__;o__;f__;g__	0	132	0.00	0.0044	0.9701
10	k__Bacteria;p__Acidobacteria;c__Acidobacteria-5;o__;f__;g__	0	131	1.00	0.0043	0.9721
11	k__Bacteria;p__Acidobacteria;c__Acidobacteria-6;o__BPC015;f__;g__	2	127	2.00	0.0045	0.9180
12	k__Bacteria;p__Acidobacteria;c__Acidobacteria-6;o__CCU21;f__;g__	2	114	23.83	0.0043	0.8022
13	k__Bacteria;p__Acidobacteria;c__Acidobacteria-6;o__iii1-15;f__;g__	0	130	2.50	0.0044	0.9578
14	k__Bacteria;p__Acidobacteria;c__Acidobacteria-6;o__iii1-15;f__RB40;g__	0	123	3.00	0.0045	0.8649
15	k__Bacteria;p__Acidobacteria;c__Acidobacteria-6;o__iii1-15;f__mb2424;g__	0	130	0.00	0.0045	0.9335
16	k__Bacteria;p__Acidobacteria;c__Acidobacteriia;o__Acidobacteriales;f__Koribacteraceae;g__	2	119	5.00	0.0043	0.8395
17	k__Bacteria;p__Acidobacteria;c__BPC102;o__MVS-40;f__;g__	0	128	26.00	0.0045	0.9058
18	k__Bacteria;p__Acidobacteria;c__DA052;o__E29;f__;g__	2	130	0.00	0.0044	0.9545
19	k__Bacteria;p__Acidobacteria;c__DA052;o__Ellin6513;f__;g__	2	123	28.00	0.0044	0.8713

20	k__Bacteria;p__Acidobacteria;c__EC1113;o__;f__;g__	2	129	36.50	0.0045	0.9274
21	k__Bacteria;p__Acidobacteria;c__PAUC37f;o__;f__;g__	0	133	1.00	0.0045	0.9569
22	k__Bacteria;p__Acidobacteria;c__RB25;o__;f__;g__	2	131	6.00	0.0044	0.9462
23	k__Bacteria;p__Acidobacteria;c__S035;o__;f__;g__	0	130	6.00	0.0044	0.9507
24	k__Bacteria;p__Acidobacteria;c__Solibacteres;o__JH-WHS99;f__;g__	2	117	10.00	0.0044	0.8145
25	k__Bacteria;p__Acidobacteria;c__Solibacteres;o__Solibacterales;f__;g__	2	119	113.83	0.0049	0.7604
26	k__Bacteria;p__Acidobacteria;c__Solibacteres;o__Solibacterales;f__AKIW659;g__	2	66	29.00	0.0039	0.4472
27	k__Bacteria;p__Acidobacteria;c__Solibacteres;o__Solibacterales;f__PAUC26f;g__	2	132	1.50	0.0044	0.9770
28	k__Bacteria;p__Acidobacteria;c__Solibacteres;o__Solibacterales;f__Solibacteraceae;g__Candidatus Solibacter	0	128	36.00	0.0046	0.8621
29	k__Bacteria;p__Acidobacteria;c__Sva0725;o__Sva0725;f__;g__	0	128	4.00	0.0045	0.9053
30	k__Bacteria;p__Acidobacteria;c__TM1;o__;f__;g__	2	121	37.00	0.0044	0.8476
31	k__Bacteria;p__Acidobacteria;c__[Chloracidobacteria];o__11-24;f__;g__	0	119	4.00	0.0044	0.8442
32	k__Bacteria;p__Acidobacteria;c__[Chloracidobacteria];o__RB41;f__;g__	0	121	1.00	0.0043	0.8772
33	k__Bacteria;p__Acidobacteria;c__[Chloracidobacteria];o__RB41;f__Ellin6075;g__	0	130	9.50	0.0045	0.9049
34	k__Bacteria;p__Acidobacteria;c__iii1-8;o__32-20;f__;g__	0	123	2.00	0.0045	0.8541
35	k__Bacteria;p__Acidobacteria;c__iii1-8;o__DS-18;f__;g__	0	131	56.00	0.0050	0.9036
36	k__Bacteria;p__Actinobacteria;c__Acidimicrobiia;o__Acidimicrobiales;f__;g__	0	132	7.00	0.0045	0.9352
37	k__Bacteria;p__Actinobacteria;c__Actinobacteria;o__Actinomycetales;f__Micrococcaceae;g__Arthrobacter	1	4	242.00	0.0027	0.0001
38	k__Bacteria;p__Actinobacteria;c__Actinobacteria;o__Actinomycetales;f__Nocardioidaceae;g__	1	5	304.00	0.0027	0.0000
39	k__Bacteria;p__Actinobacteria;c__MB-A2-108;o__;f__;g__	2	126	1.00	0.0043	0.9254
40	k__Bacteria;p__Actinobacteria;c__MB-A2-108;o__0319-7L14;f__;g__	0	90	68.50	0.0043	0.5801
41	k__Bacteria;p__Actinobacteria;c__Rubrobacteria;o__Rubrobacterales;f__Rubrobacteraceae;g__	0	110	2.00	0.0043	0.7590
42	k__Bacteria;p__Actinobacteria;c__Thermoleophilia;o__Gaiellales;f__Gaiellaceae;g__	0	125	7.00	0.0045	0.8747
43	k__Bacteria;p__Actinobacteria;c__Thermoleophilia;o__Solirubrobacterales;f__;g__	0	105	44.25	0.0047	0.6395
44	k__Bacteria;p__Armatimonadetes;c__0319-6E2;o__;f__;g__	2	107	125.25	0.0047	0.6948
45	k__Bacteria;p__Armatimonadetes;c__[Fimbriimonadia];o__[Fimbriimonadales];f__;g__	0	131	18.00	0.0046	0.8893

46	k__Bacteria;p__Bacteroidetes;c__Cytophagia;o__Cytophagales;f__Cytophagaceae;g__	1	8	3534.00	0.0031	0.0007
47	k__Bacteria;p__Bacteroidetes;c__Flavobacteriia;o__Flavobacteriales;f__Cryomorphaceae;g__Fluviicola	2	89	16.00	0.0042	0.5643
48	k__Bacteria;p__Bacteroidetes;c__Flavobacteriia;o__Flavobacteriales;f__Flavobacteriaceae;g__Flavobacterium	0	81	81.00	0.0041	0.5079
49	k__Bacteria;p__Bacteroidetes;c__Sphingobacteriia;o__Sphingobacteriales;f__;g__	2	121	281.42	0.0050	0.7873
50	k__Bacteria;p__Bacteroidetes;c__[Saprospirae];o__[Saprospirales];f__Chitinophagaceae;g__	0	115	12.00	0.0044	0.7681
51	k__Bacteria;p__Bacteroidetes;c__[Saprospirae];o__[Saprospirales];f__Chitinophagaceae;g__Flavisolibacter	0	42	2.00	0.0038	0.2460
52	k__Bacteria;p__Bacteroidetes;c__[Saprospirae];o__[Saprospirales];f__Chitinophagaceae;g__Sediminibacterium	1	1	0.00	0.0020	0.0000
53	k__Bacteria;p__Bacteroidetes;c__[Saprospirae];o__[Saprospirales];f__Saprospiraceae;g__	0	124	21.00	0.0045	0.8402
54	k__Bacteria;p__Chlorobi;c__;o__;f__;g__	0	114	43.75	0.0044	0.7706
55	k__Bacteria;p__Chlorobi;c__BSV26;o__A89;f__;g__	2	130	11.00	0.0044	0.9570
56	k__Bacteria;p__Chlorobi;c__BSV26;o__C20;f__;g__	0	130	3.00	0.0046	0.9016
57	k__Bacteria;p__Chlorobi;c__BSV26;o__PK329;f__;g__	0	119	3.00	0.0044	0.8682
58	k__Bacteria;p__Chlorobi;c__BSV26;o__VC38;f__;g__	0	36	252.50	0.0038	0.1988
59	k__Bacteria;p__Chlorobi;c__SJA-28;o__;f__;g__	2	132	4.00	0.0045	0.9714
60	k__Bacteria;p__Chloroflexi;c__Anaerolineae;o__A31;f__;g__	0	118	196.00	0.0045	0.7960
61	k__Bacteria;p__Chloroflexi;c__Anaerolineae;o__CFB-26;f__;g__	0	135	74.75	0.0050	0.9517
62	k__Bacteria;p__Chloroflexi;c__Anaerolineae;o__GCA004;f__;g__	2	130	0.00	0.0045	0.9324
63	k__Bacteria;p__Chloroflexi;c__Anaerolineae;o__H39;f__;g__	2	133	8.00	0.0044	0.9817
64	k__Bacteria;p__Chloroflexi;c__Anaerolineae;o__S0208;f__;g__	2	128	2.00	0.0045	0.9267
65	k__Bacteria;p__Chloroflexi;c__Anaerolineae;o__SB-34;f__;g__	2	129	10.00	0.0046	0.8941
66	k__Bacteria;p__Chloroflexi;c__Anaerolineae;o__SBR1031;f__A4b;g__	2	116	253.25	0.0048	0.8022
67	k__Bacteria;p__Chloroflexi;c__Anaerolineae;o__envOPS12;f__;g__	0	136	4.00	0.0045	1.0000
68	k__Bacteria;p__Chloroflexi;c__Anaerolineae;o__pLW-97;f__;g__	2	128	7.75	0.0044	0.9260
69	k__Bacteria;p__Chloroflexi;c__Chloroflexi;o__[Roseiflexales];f__[Kouleothrixaceae];g__	2	32	0.00	0.0035	0.1929
70	k__Bacteria;p__Chloroflexi;c__Ellin6529;o__;f__;g__	2	119	390.25	0.0050	0.7470
71	k__Bacteria;p__Chloroflexi;c__Gitt-GS-136;o__;f__;g__	0	88	112.25	0.0044	0.5696

72	k__Bacteria;p__Chloroflexi;c__P2-11E;o__;f__;g__	0	127	2.00	0.0045	0.8856
73	k__Bacteria;p__Chloroflexi;c__S085;o__;f__;g__	2	125	8.00	0.0044	0.9081
74	k__Bacteria;p__Chloroflexi;c__SAR202;o__;f__;g__	2	130	233.00	0.0048	0.9415
75	k__Bacteria;p__Chloroflexi;c__TK10;o__AKYG885;f__5B-12;g__	2	130	9.25	0.0045	0.9315
76	k__Bacteria;p__Firmicutes;c__Bacilli;o__Bacillales;f__Bacillaceae;g__Bacillus	0	128	0.00	0.0044	0.9448
77	k__Bacteria;p__Firmicutes;c__Bacilli;o__Bacillales;f__Paenibacillaceae;g__Paenibacillus	0	60	295.75	0.0039	0.3611
78	k__Bacteria;p__Firmicutes;c__Bacilli;o__Bacillales;f__Planococcaceae;g__Sporosarcina	1	4	0.00	0.0032	0.0058
79	k__Bacteria;p__Firmicutes;c__Bacilli;o__Lactobacillales;f__Leuconostocaceae;g__Leuconostoc	0	118	4.50	0.0044	0.8119
80	k__Bacteria;p__Firmicutes;c__Bacilli;o__Lactobacillales;f__Streptococcaceae;g__Lactococcus	0	127	16.00	0.0045	0.9028
81	k__Bacteria;p__Firmicutes;c__Bacilli;o__Lactobacillales;f__Streptococcaceae;g__Streptococcus	0	111	80.00	0.0045	0.7075
82	k__Bacteria;p__Firmicutes;c__Clostridia;o__Clostridiales;f__Peptococcaceae;g__Desulfosporosinus	1	13	172.00	0.0020	0.0000
83	k__Bacteria;p__GAL15;c__;o__;f__;g__	2	128	4.50	0.0045	0.9252
84	k__Bacteria;p__GN04;c__MSB-5A5;o__;f__;g__	2	134	5.00	0.0045	0.9730
85	k__Bacteria;p__Gemmatimonadetes;c__;o__;f__;g__	0	112	30.50	0.0045	0.7400
86	k__Bacteria;p__Gemmatimonadetes;c__Gemm-1;o__;f__;g__	0	130	0.00	0.0044	0.9567
87	k__Bacteria;p__Gemmatimonadetes;c__Gemm-2;o__;f__;g__	2	133	23.00	0.0045	0.9662
88	k__Bacteria;p__Gemmatimonadetes;c__Gemm-5;o__;f__;g__	0	129	7.00	0.0045	0.9034
89	k__Bacteria;p__Gemmatimonadetes;c__Gemmatimonadetes;o__;f__;g__	0	115	117.25	0.0048	0.7530
90	k__Bacteria;p__Gemmatimonadetes;c__Gemmatimonadetes;o__C114;f__;g__	0	124	1.00	0.0045	0.8762
91	k__Bacteria;p__NC10;c__12-24;Other;Other;Other	2	129	1.00	0.0044	0.9551
92	k__Bacteria;p__NC10;c__12-24;o__JH-WHS47;f__;g__	2	120	63.50	0.0044	0.8432
93	k__Bacteria;p__NC10;c__12-24;o__MIZ17;f__;g__	2	102	129.00	0.0043	0.6839
94	k__Bacteria;p__NC10;c__wb1-A12;o__;f__;g__	2	129	5.00	0.0044	0.9629
95	k__Bacteria;p__Nitrospirae;c__Nitrospira;o__Nitrospirales;f__0319-6A21;g__	2	126	0.00	0.0044	0.9230
96	k__Bacteria;p__Nitrospirae;c__Nitrospira;o__Nitrospirales;f__Nitrospiraceae;g__	2	130	0.00	0.0044	0.9569
97	k__Bacteria;p__Nitrospirae;c__Nitrospira;o__Nitrospirales;f__Nitrospiraceae;g__JG37-AG-70	2	131	9.00	0.0044	0.9593

98	k__Bacteria;p__Nitrospirae;c__Nitrospira;o__Nitrospirales;f__Nitrospiraceae;g__Nitrospira	2	87	45.00	0.0043	0.5259
99	k__Bacteria;p__Nitrospirae;c__Nitrospira;o__Nitrospirales;f__[Leptospirillaceae];g__	2	122	15.00	0.0044	0.8732
100	k__Bacteria;p__OP3;c__PBS-25;o__;f__;g__	0	126	4.00	0.0044	0.9012
101	k__Bacteria;p__OP3;c__koll11;o__;f__;g__	0	132	0.00	0.0044	0.9640
102	k__Bacteria;p__OP3;c__koll11;o__GIF10;f__kpj58rc;g__	0	93	65.50	0.0043	0.6009
103	k__Bacteria;p__Planctomycetes;Other;Other;Other;Other	2	131	1.00	0.0045	0.9624
104	k__Bacteria;p__Planctomycetes;c__OM190;o__CL500-15;f__;g__	0	130	4.00	0.0044	0.9247
105	k__Bacteria;p__Planctomycetes;c__OM190;o__agg27;f__;g__	0	132	5.00	0.0045	0.9469
106	k__Bacteria;p__Planctomycetes;c__Phycisphaerae;o__CCM11a;f__;g__	2	125	13.50	0.0044	0.8990
107	k__Bacteria;p__Planctomycetes;c__Phycisphaerae;o__Phycisphaerales;f__;g__	0	130	1.50	0.0043	0.9703
108	k__Bacteria;p__Planctomycetes;c__Phycisphaerae;o__Phycisphaerales;f__Phycisphaeraceae;g__	2	131	11.00	0.0045	0.9491
109	k__Bacteria;p__Planctomycetes;c__Phycisphaerae;o__WD2101;f__;g__	0	128	4.00	0.0044	0.8985
110	k__Bacteria;p__Planctomycetes;c__Pla3;o__;f__;g__	2	132	0.00	0.0045	0.9524
111	k__Bacteria;p__Planctomycetes;c__Planctomycetia;o__Gemmatales;f__Gemmataceae;g__	2	133	3.00	0.0045	0.9789
112	k__Bacteria;p__Planctomycetes;c__Planctomycetia;o__Gemmatales;f__Gemmataceae;g__Gemmata	0	116	14.75	0.0044	0.8100
113	k__Bacteria;p__Planctomycetes;c__Planctomycetia;o__Gemmatales;f__Isosphaeraceae;g__	2	133	1.00	0.0044	0.9805
114	k__Bacteria;p__Planctomycetes;c__Planctomycetia;o__Pirellulales;f__Pirellulaceae;g__	0	133	0.00	0.0044	0.9848
115	k__Bacteria;p__Planctomycetes;c__Planctomycetia;o__Pirellulales;f__Pirellulaceae;g__A17	0	70	38.00	0.0040	0.4269
116	k__Bacteria;p__Planctomycetes;c__Planctomycetia;o__Pirellulales;f__Pirellulaceae;g__Pirellula	0	134	9.00	0.0045	0.9472
117	k__Bacteria;p__Planctomycetes;c__Planctomycetia;o__Planctomycetales;f__Planctomycetaceae;g__Planctomyces	0	119	28.00	0.0049	0.7783
118	k__Bacteria;p__Proteobacteria;c__;o__;f__;g__	0	131	5.00	0.0045	0.9340
119	k__Bacteria;p__Proteobacteria;c__Alphaproteobacteria;o__Caulobacterales;f__Caulobacteraceae;g__	1	7	2.00	0.0017	0.0000
120	k__Bacteria;p__Proteobacteria;c__Alphaproteobacteria;o__Caulobacterales;f__Caulobacteraceae;g__Mycoplana	1	17	675.00	0.0020	0.0000
121	k__Bacteria;p__Proteobacteria;c__Alphaproteobacteria;o__Ellin329;f__;g__	0	134	19.00	0.0045	0.9679
122	k__Bacteria;p__Proteobacteria;c__Alphaproteobacteria;o__Rhizobiales;f__;g__	2	96	55.00	0.0043	0.5977
123	k__Bacteria;p__Proteobacteria;c__Alphaproteobacteria;o__Rhizobiales;f__Bradyrhizobiaceae;g__Bradyrhizobium	1	11	359.00	0.0025	0.0000

124	k__Bacteria;p__Proteobacteria;c__Alphaproteobacteria;o__Rhizobiales;f__Hyphomicrobiaceae;g__	1	6	291.00	0.0034	0.0145
125	k__Bacteria;p__Proteobacteria;c__Alphaproteobacteria;o__Rhizobiales;f__Hyphomicrobiaceae;g__Devosia	1	14	178.00	0.0020	0.0000
126	k__Bacteria;p__Proteobacteria;c__Alphaproteobacteria;o__Rhizobiales;f__Hyphomicrobiaceae;g__Rhodoplanes	1	5	758.00	0.0038	0.0155
127	k__Bacteria;p__Proteobacteria;c__Alphaproteobacteria;o__Rhodospirillales;f__;g__	0	111	191.75	0.0044	0.7379
128	k__Bacteria;p__Proteobacteria;c__Alphaproteobacteria;o__Rhodospirillales;f__Rhodospirillaceae;g__	2	69	879.00	0.0042	0.3958
129	k__Bacteria;p__Proteobacteria;c__Alphaproteobacteria;o__Rhodospirillales;f__Rhodospirillaceae;g__Magnetospirillum	1	7	5.00	0.0017	0.0000
130	k__Bacteria;p__Proteobacteria;c__Alphaproteobacteria;o__Sphingomonadales;f__Sphingomonadaceae;g__Sphingomonas	1	2	0.00	0.0025	0.0000
131	k__Bacteria;p__Proteobacteria;c__Betaproteobacteria;o__;f__;g__	2	128	13.00	0.0044	0.9401
132	k__Bacteria;p__Proteobacteria;c__Betaproteobacteria;o__Burkholderiales;f__Comamonadaceae;Other	1	11	18.25	0.0020	0.0000
133	k__Bacteria;p__Proteobacteria;c__Betaproteobacteria;o__Burkholderiales;f__Comamonadaceae;g__Hydrogenophaga	1	10	190.25	0.0017	0.0000
134	k__Bacteria;p__Proteobacteria;c__Betaproteobacteria;o__Burkholderiales;f__Comamonadaceae;g__Limnohabitans	1	12	324.00	0.0020	0.0000
135	k__Bacteria;p__Proteobacteria;c__Betaproteobacteria;o__Burkholderiales;f__Comamonadaceae;g__Methylibium	1	6	4.00	0.0017	0.0000
136	k__Bacteria;p__Proteobacteria;c__Betaproteobacteria;o__Burkholderiales;f__Comamonadaceae;g__Polaromonas	1	3	2.00	0.0015	0.0000
137	k__Bacteria;p__Proteobacteria;c__Betaproteobacteria;o__Burkholderiales;f__Comamonadaceae;g__Variovorax	1	3	0.00	0.0020	0.0000
138	k__Bacteria;p__Proteobacteria;c__Betaproteobacteria;o__Ellin6067;f__;g__	1	6	599.50	0.0025	0.0000
139	k__Bacteria;p__Proteobacteria;c__Betaproteobacteria;o__Gallionellales;f__Gallionellaceae;g__Gallionella	1	7	1182.00	0.0025	0.0000
140	k__Bacteria;p__Proteobacteria;c__Betaproteobacteria;o__IS-44;f__;g__	2	60	414.50	0.0041	0.3557
141	k__Bacteria;p__Proteobacteria;c__Betaproteobacteria;o__MND1;f__;g__	2	131	9.00	0.0045	0.9326
142	k__Bacteria;p__Proteobacteria;c__Betaproteobacteria;o__Methylophilales;f__Methylophilaceae;g__	2	4	0.00	0.0035	0.0146
143	k__Bacteria;p__Proteobacteria;c__Betaproteobacteria;o__Nitrosomonadales;f__Nitrosomonadaceae;g__	2	1	0.00	0.0028	0.0005
144	k__Bacteria;p__Proteobacteria;c__Betaproteobacteria;o__Rhodocyclales;f__Rhodocyclaceae;g__Azoarcus	2	29	1.00	0.0036	0.1673
145	k__Bacteria;p__Proteobacteria;c__Betaproteobacteria;o__Rhodocyclales;f__Rhodocyclaceae;g__Dechloromonas	1	10	8.00	0.0017	0.0000
146	k__Bacteria;p__Proteobacteria;c__Betaproteobacteria;o__Rhodocyclales;f__Rhodocyclaceae;g__Dok59	1	8	4.00	0.0020	0.0000
147	k__Bacteria;p__Proteobacteria;c__Betaproteobacteria;o__Rhodocyclales;f__Rhodocyclaceae;g__Sulfuritalea	1	12	1.00	0.0020	0.0000
148	k__Bacteria;p__Proteobacteria;c__Betaproteobacteria;o__SBla14;f__;g__	1	3	0.00	0.0016	0.0000
149	k__Bacteria;p__Proteobacteria;c__Deltaproteobacteria;o__;f__;g__	2	47	2.00	0.0037	0.2837

150	k__Bacteria;p__Proteobacteria;c__Deltaproteobacteria;o__DTB120;f__;g__	1	2	7.00	0.0019	0.0000
151	k__Bacteria;p__Proteobacteria;c__Deltaproteobacteria;o__Desulfobacterales;f__Desulfobulbaceae;g__	1	8	0.00	0.0017	0.0000
152	k__Bacteria;p__Proteobacteria;c__Deltaproteobacteria;o__Myxococcales;f__;g__	2	116	400.25	0.0049	0.7205
153	k__Bacteria;p__Proteobacteria;c__Deltaproteobacteria;o__Myxococcales;f__Haliangiaceae;g__	0	130	33.00	0.0045	0.9149
154	k__Bacteria;p__Proteobacteria;c__Deltaproteobacteria;o__NB1-j;f__;g__	2	109	12.00	0.0044	0.6997
155	k__Bacteria;p__Proteobacteria;c__Deltaproteobacteria;o__NB1-j;f__NB1-i;g__	2	133	2.00	0.0044	0.9938
156	k__Bacteria;p__Proteobacteria;c__Deltaproteobacteria;o__Syntrophobacterales;f__Syntrophobacteraceae;g__	2	132	13.00	0.0044	0.9752
157	k__Bacteria;p__Proteobacteria;c__Deltaproteobacteria;o__[Entotheonellales];f__[Entotheonellaceae];g__	0	128	0.00	0.0044	0.9248
158	k__Bacteria;p__Proteobacteria;c__Gammaproteobacteria;Other;Other;Other	2	109	20.00	0.0043	0.7553
159	k__Bacteria;p__Proteobacteria;c__Gammaproteobacteria;o__Alteromonadales;f__Alteromonadaceae;g__Cellvibrio	0	1	0.00	0.0030	0.0050
160	k__Bacteria;p__Proteobacteria;c__Gammaproteobacteria;o__Chromatiales;f__Ectothiorhodospiraceae;g__	0	119	42.00	0.0045	0.8144
161	k__Bacteria;p__Proteobacteria;c__Gammaproteobacteria;o__Legionellales;f__Coxiellaceae;g__Aquicella	0	96	23.00	0.0043	0.6205
162	k__Bacteria;p__Proteobacteria;c__Gammaproteobacteria;o__Pseudomonadales;f__Pseudomonadaceae;g__Pseudomonas	1	1	0.00	0.0021	0.0000
163	k__Bacteria;p__Proteobacteria;c__Gammaproteobacteria;o__Thiotrichales;f__Piscirickettsiaceae;g__	0	127	23.00	0.0046	0.8613
164	k__Bacteria;p__Proteobacteria;c__Gammaproteobacteria;o__Xanthomonadales;f__Sinobacteraceae;g__	0	132	3.00	0.0043	0.9683
165	k__Bacteria;p__Proteobacteria;c__Gammaproteobacteria;o__Xanthomonadales;f__Sinobacteraceae;g__Steroidobacter	0	114	7.25	0.0044	0.7832
166	k__Bacteria;p__Proteobacteria;c__Gammaproteobacteria;o__Xanthomonadales;f__Xanthomonadaceae;g__	1	10	1131.00	0.0025	0.0000
167	k__Bacteria;p__Proteobacteria;c__Gammaproteobacteria;o__Xanthomonadales;f__Xanthomonadaceae;g__Luteimonas	1	9	11.00	0.0020	0.0000
168	k__Bacteria;p__Proteobacteria;c__Gammaproteobacteria;o__Xanthomonadales;f__Xanthomonadaceae;g__Lysobacter	1	14	2.00	0.0020	0.0000
169	k__Bacteria;p__Proteobacteria;c__Gammaproteobacteria;o__Xanthomonadales;f__Xanthomonadaceae;g__Thermomonas	1	13	1.00	0.0020	0.0000
170	k__Bacteria;p__SBR1093;c__;o__;f__;g__	2	129	8.50	0.0045	0.9238
171	k__Bacteria;p__Verrucomicrobia;c__Opitutae;o__Opitutales;f__Opitutaceae;g__	1	4	446.00	0.0033	0.0023
172	k__Bacteria;p__Verrucomicrobia;c__Opitutae;o__Opitutales;f__Opitutaceae;g__Opitutus	2	21	2969.50	0.0041	0.0837
173	k__Bacteria;p__WS3;c__PRR-12;o__LD1-PA13;f__;g__	2	132	11.50	0.0045	0.9731
174	k__Bacteria;p__WS3;c__PRR-12;o__Sediment-1;f__;g__	0	129	1.00	0.0043	0.9761
175	k__Bacteria;p__WS3;c__PRR-12;o__Sediment-1;f__PRR-10;g__	2	131	1.50	0.0044	0.9748

176	k__Bacteria;p__WS3;c__PRR-12;o__wb1_H11;f__;g__	2	120	8.00	0.0043	0.8720
177	k__Bacteria;p__[Caldithrix];c__KSB1;o__Ucn15732;f__;g__	2	122	69.17	0.0045	0.8285

Supplementary Table 11 nodes and edges information of 10 cm co-occurrence network

Id	name	modularity_class	degree	betweenness	closeness	eigenvector
0	k__Archaea;p__Crenarchaeota;c__Thaumarchaeota;o__AK31;f__;g__	2	31	143	0.0035	0.2872
1	k__Archaea;p__Crenarchaeota;c__Thaumarchaeota;o__Cenarchaeales;f__Cenarchaeaceae;g__	1	21	20	0.0035	0.1680
2	k__Archaea;p__Crenarchaeota;c__Thaumarchaeota;o__Cenarchaeales;f__Cenarchaeaceae;g__Nitrosopumilus	1	17	98	0.0034	0.0094
3	k__Archaea;p__Crenarchaeota;c__Thaumarchaeota;o__Cenarchaeales;f__SAGMA-X;g__	2	68	6	0.0036	0.7442
4	k__Archaea;p__Crenarchaeota;c__Thaumarchaeota;o__Nitrososphaerales;f__Nitrososphaeraceae;g__Candidatus Nitrososphaera	1	16	67	0.0034	0.0071
5	k__Archaea;p__Euryarchaeota;c__Thermoplasmata;o__E2;f__;g__	2	80	83	0.0043	0.7693
6	k__Archaea;p__Euryarchaeota;c__Thermoplasmata;o__E2;f__[Methanomassiliicoccaceae];g__	2	72	3	0.0038	0.8422
7	k__Archaea;p__[Parvarchaeota];c__[Parvarchaea];o__YLA114;f__;g__	2	70	9	0.0038	0.7713
8	k__Bacteria;Other;Other;Other;Other;Other	2	83	9.5	0.0043	0.8550
9	k__Bacteria;p__Acidobacteria;c__;o__;f__;g__	2	84	31	0.0043	0.9370
10	k__Bacteria;p__Acidobacteria;c__Acidobacteria-5;o__;f__;g__	1	101	207.75	0.0046	0.8913
11	k__Bacteria;p__Acidobacteria;c__Acidobacteria-6;o__BPC015;f__;g__	2	76	2	0.0039	0.9034
12	k__Bacteria;p__Acidobacteria;c__Acidobacteria-6;o__CCU21;f__;g__	2	78	6	0.0039	0.9112
13	k__Bacteria;p__Acidobacteria;c__Acidobacteria-6;o__iii1-15;f__;g__	1	90	537.25	0.0046	0.6954
14	k__Bacteria;p__Acidobacteria;c__Acidobacteria-6;o__iii1-15;f__RB40;g__	1	58	277.25	0.0045	0.2363
15	k__Bacteria;p__Acidobacteria;c__Acidobacteria-6;o__iii1-15;f__mb2424;g__	1	57	41	0.0043	0.2358
16	k__Bacteria;p__Acidobacteria;c__Acidobacteriia;o__Acidobacteriales;f__Koribacteraceae;g__	2	82	11	0.0040	0.9318
17	k__Bacteria;p__Acidobacteria;c__BPC102;o__MVS-40;f__;g__	2	88	277.75	0.0045	0.8408

18	k__Bacteria;p__Acidobacteria;c__DA052;o__E29;f__;g__	2	71	0	0.0038	0.8622
19	k__Bacteria;p__Acidobacteria;c__DA052;o__Ellin6513;f__;g__	2	68	1	0.0038	0.8359
20	k__Bacteria;p__Acidobacteria;c__EC1113;o__;f__;g__	2	80	0	0.0039	0.9303
21	k__Bacteria;p__Acidobacteria;c__PAUC37f;o__;f__;g__	1	30	291.5	0.0038	0.2295
22	k__Bacteria;p__Acidobacteria;c__RB25;o__;f__;g__	2	83	0	0.0040	0.9553
23	k__Bacteria;p__Acidobacteria;c__S035;o__;f__;g__	1	60	83.5	0.0042	0.3540
24	k__Bacteria;p__Acidobacteria;c__Solibacteres;o__JH-WHS99;f__;g__	2	76	4	0.0039	0.8987
25	k__Bacteria;p__Acidobacteria;c__Solibacteres;o__Solibacterales;f__;g__	1	37	95	0.0039	0.2822
26	k__Bacteria;p__Acidobacteria;c__Solibacteres;o__Solibacterales;f__AKIW659;g__	2	75	4	0.0039	0.8909
27	k__Bacteria;p__Acidobacteria;c__Solibacteres;o__Solibacterales;f__PAUC26f;g__	2	78	0	0.0039	0.9349
28	k__Bacteria;p__Acidobacteria;c__Solibacteres;o__Solibacterales;f__Solibacteraceae;g__Candidatus Solibacter	1	22	31	0.0038	0.0758
29	k__Bacteria;p__Acidobacteria;c__Sva0725;o__Sva0725;f__;g__	1	52	484.5	0.0044	0.1829
30	k__Bacteria;p__Acidobacteria;c__TM1;o__;f__;g__	2	82	2	0.0039	0.9435
31	k__Bacteria;p__Acidobacteria;c__[Chloracidobacteria];o__11-24;f__;g__	2	77	177	0.0043	0.6956
32	k__Bacteria;p__Acidobacteria;c__[Chloracidobacteria];o__RB41;f__;g__	1	35	170	0.0038	0.0534
33	k__Bacteria;p__Acidobacteria;c__[Chloracidobacteria];o__RB41;f__Ellin6075;g__	1	27	12	0.0035	0.0290
34	k__Bacteria;p__Acidobacteria;c__iii1-8;o__32-20;f__;g__	1	51	65	0.0042	0.2236
35	k__Bacteria;p__Acidobacteria;c__iii1-8;o__DS-18;f__;g__	1	47	5	0.0042	0.1676
36	k__Bacteria;p__Actinobacteria;c__Acidimicrobiia;o__Acidimicrobiales;f__;g__	1	38	14	0.0041	0.0952
37	k__Bacteria;p__Actinobacteria;c__Actinobacteria;o__Actinomycetales;f__Micrococcaceae;g__Arth robacter	0	5	168	0.0018	0.0000
38	k__Bacteria;p__Actinobacteria;c__Actinobacteria;o__Actinomycetales;f__Nocardioidaceae;g__	0	9	1887	0.0024	0.0000
39	k__Bacteria;p__Actinobacteria;c__MB-A2-108;o__;f__;g__	2	84	55	0.0041	0.8961
40	k__Bacteria;p__Actinobacteria;c__MB-A2-108;o__0319-7L14;f__;g__	1	49	131	0.0041	0.2780
41	k__Bacteria;p__Actinobacteria;c__Rubrobacteria;o__Rubrobacterales;f__Rubrobacteraceae;g__	1	4	34	0.0027	0.0004

42	k__Bacteria;p__Actinobacteria;c__Thermoleophilia;o__Gaiellales;f__Gaiellaceae;g__	2	95	302.75	0.0045	0.8623
43	k__Bacteria;p__Actinobacteria;c__Thermoleophilia;o__Solirubrobacterales;f__;g__	1	33	11	0.0039	0.2190
44	k__Bacteria;p__Armatimonadetes;c__0319-6E2;o__;f__;g__	1	64	80.5	0.0042	0.5198
45	k__Bacteria;p__Armatimonadetes;c__[Fimbriimonadia];o__[Fimbriimonadales];f__;g__	1	42	208	0.0043	0.1337
46	k__Bacteria;p__Bacteroidetes;c__Cytophagia;o__Cytophagales;f__Cytophagaceae;g__	1	18	58	0.0033	0.0130
47	k__Bacteria;p__Bacteroidetes;c__Flavobacteriia;o__Flavobacteriales;f__Cryomorphaceae;g__Fluvii cola	1	4	3	0.0027	0.0004
48	k__Bacteria;p__Bacteroidetes;c__Flavobacteriia;o__Flavobacteriales;f__Flavobacteriaceae;g__Flav obacterium	0	1	0	0.0015	0.0000
49	k__Bacteria;p__Bacteroidetes;c__Sphingobacteriia;o__Sphingobacteriales;f__;g__	1	2	0	0.0026	0.0001
50	k__Bacteria;p__Bacteroidetes;c__[Saprospirae];o__[Saprospirales];f__Chitinophagaceae;g__	1	33	2277	0.0039	0.0413
51	k__Bacteria;p__Bacteroidetes;c__[Saprospirae];o__[Saprospirales];f__Chitinophagaceae;g__Flaviso libacter	1	5	0	0.0030	0.0023
52	k__Bacteria;p__Bacteroidetes;c__[Saprospirae];o__[Saprospirales];f__Chitinophagaceae;g__Sedimi nibacterium	0	9	0	0.0019	0.0000
53	k__Bacteria;p__Bacteroidetes;c__[Saprospirae];o__[Saprospirales];f__Saprospiraceae;g__	1	41	114	0.0040	0.0847
54	k__Bacteria;p__Chlorobi;c__;o__;f__;g__	1	25	3	0.0035	0.0311
55	k__Bacteria;p__Chlorobi;c__BSV26;o__A89;f__;g__	2	66	64.25	0.0035	0.8005
56	k__Bacteria;p__Chlorobi;c__BSV26;o__C20;f__;g__	2	77	19	0.0039	0.8259
57	k__Bacteria;p__Chlorobi;c__BSV26;o__PK329;f__;g__	2	22	50.5	0.0036	0.1912
58	k__Bacteria;p__Chlorobi;c__BSV26;o__VC38;f__;g__	1	20	298	0.0036	0.0142
59	k__Bacteria;p__Chlorobi;c__SJA-28;o__;f__;g__	2	72	0	0.0038	0.8574
60	k__Bacteria;p__Chloroflexi;c__Anaerolineae;o__A31;f__;g__	2	57	0	0.0035	0.6765
61	k__Bacteria;p__Chloroflexi;c__Anaerolineae;o__CFB-26;f__;g__	1	83	322	0.0045	0.6304
62	k__Bacteria;p__Chloroflexi;c__Anaerolineae;o__GCA004;f__;g__	2	91	6	0.0041	0.9500
63	k__Bacteria;p__Chloroflexi;c__Anaerolineae;o__H39;f__;g__	2	79	0	0.0039	0.9474

64	k__Bacteria;p__Chloroflexi;c__Anaerolineae;o__S0208;f__;g__	2	73	0	0.0037	0.8953
65	k__Bacteria;p__Chloroflexi;c__Anaerolineae;o__SB-34;f__;g__	2	73	2	0.0038	0.8749
66	k__Bacteria;p__Chloroflexi;c__Anaerolineae;o__SBR1031;f__A4b;g__	2	76	1	0.0038	0.8769
67	k__Bacteria;p__Chloroflexi;c__Anaerolineae;o__envOPS12;f__;g__	1	34	633.5	0.0042	0.1326
68	k__Bacteria;p__Chloroflexi;c__Anaerolineae;o__pLW-97;f__;g__	2	77	4	0.0038	0.9256
69	k__Bacteria;p__Chloroflexi;c__Chloroflexi;o__[Roseiflexales];f__[Kouleothrixaceae];g__	2	78	48	0.0041	0.8318
70	k__Bacteria;p__Chloroflexi;c__Ellin6529;o__;f__;g__	1	40	65.5	0.0039	0.2164
71	k__Bacteria;p__Chloroflexi;c__Gitt-GS-136;o__;f__;g__	1	33	106	0.0039	0.0700
72	k__Bacteria;p__Chloroflexi;c__P2-11E;o__;f__;g__	1	38	79	0.0041	0.1273
73	k__Bacteria;p__Chloroflexi;c__S085;o__;f__;g__	2	86	3	0.0040	0.9695
74	k__Bacteria;p__Chloroflexi;c__SAR202;o__;f__;g__	2	85	6	0.0040	0.9752
75	k__Bacteria;p__Chloroflexi;c__TK10;o__AKYG885;f__5B-12;g__	2	79	0	0.0039	0.9322
76	k__Bacteria;p__Firmicutes;c__Bacilli;o__Bacillales;f__Bacillaceae;g__Bacillus	2	92	123.5	0.0044	0.8497
77	k__Bacteria;p__Firmicutes;c__Bacilli;o__Bacillales;f__Paenibacillaceae;g__Paenibacillus	1	2	37	0.0025	0.0000
78	k__Bacteria;p__Firmicutes;c__Bacilli;o__Bacillales;f__Planococcaceae;g__Sporosarcina	1	14	745	0.0032	0.0026
79	k__Bacteria;p__Firmicutes;c__Bacilli;o__Lactobacillales;f__Leuconostocaceae;g__Leuconostoc	2	92	18	0.0045	0.9013
80	k__Bacteria;p__Firmicutes;c__Bacilli;o__Lactobacillales;f__Streptococcaceae;g__Lactococcus	2	87	19	0.0043	0.8989
81	k__Bacteria;p__Firmicutes;c__Bacilli;o__Lactobacillales;f__Streptococcaceae;g__Streptococcus	2	95	90	0.0045	0.9557
82	k__Bacteria;p__GAL15;c__;o__;f__;g__	2	89	13	0.0041	0.9469
83	k__Bacteria;p__GN04;c__MSB-5A5;o__;f__;g__	2	72	11	0.0038	0.8353
84	k__Bacteria;p__Gemmatimonadetes;c__;o__;f__;g__	1	51	40	0.0042	0.1564
85	k__Bacteria;p__Gemmatimonadetes;c__Gemm-1;o__;f__;g__	2	98	62.25	0.0045	0.9213
86	k__Bacteria;p__Gemmatimonadetes;c__Gemm-2;o__;f__;g__	1	97	408.25	0.0046	0.7804
87	k__Bacteria;p__Gemmatimonadetes;c__Gemm-5;o__;f__;g__	1	68	406.25	0.0043	0.4221
88	k__Bacteria;p__Gemmatimonadetes;c__Gemmatimonadetes;o__;f__;g__	1	74	164.25	0.0043	0.4904
89	k__Bacteria;p__Gemmatimonadetes;c__Gemmatimonadetes;o__C114;f__;g__	1	55	77	0.0044	0.2588

90	k__Bacteria;p__NC10;c__12-24;Other;Other;Other	2	70	6	0.0038	0.8501
91	k__Bacteria;p__NC10;c__12-24;o__JH-WHS47;f__;g__	2	75	0	0.0039	0.9069
92	k__Bacteria;p__NC10;c__12-24;o__MIZ17;f__;g__	2	76	1	0.0038	0.9149
93	k__Bacteria;p__NC10;c__wb1-A12;o__;f__;g__	2	76	0	0.0039	0.9170
94	k__Bacteria;p__Nitrospirae;c__Nitrospira;o__Nitrospirales;f__0319-6A21;g__	2	84	2	0.0040	1.0000
95	k__Bacteria;p__Nitrospirae;c__Nitrospira;o__Nitrospirales;f__Nitrospiraceae;g__	2	79	0	0.0038	0.9641
96	k__Bacteria;p__Nitrospirae;c__Nitrospira;o__Nitrospirales;f__Nitrospiraceae;g__JG37-AG-70	2	67	6	0.0038	0.7455
97	k__Bacteria;p__Nitrospirae;c__Nitrospira;o__Nitrospirales;f__Nitrospiraceae;g__Nitrospira	1	80	345.75	0.0044	0.6007
98	k__Bacteria;p__Nitrospirae;c__Nitrospira;o__Nitrospirales;f__[Leptospirillaceae];g__	2	66	3	0.0036	0.8094
99	k__Bacteria;p__OP3;c__PBS-25;o__;f__;g__	1	15	245.5	0.0036	0.0346
100	k__Bacteria;p__OP3;c__koll11;o__;f__;g__	2	80	125.5	0.0041	0.8090
101	k__Bacteria;p__OP3;c__koll11;o__GIF10;f__kpj58rc;g__	1	5	25	0.0030	0.0009
102	k__Bacteria;p__Planctomycetes;Other;Other;Other;Other	2	79	0	0.0039	0.9327
103	k__Bacteria;p__Planctomycetes;c__OM190;o__CL500-15;f__;g__	2	73	93.5	0.0042	0.6904
104	k__Bacteria;p__Planctomycetes;c__OM190;o__agg27;f__;g__	1	60	124.5	0.0042	0.4547
105	k__Bacteria;p__Planctomycetes;c__Phycisphaerae;o__CCM11a;f__;g__	2	78	3	0.0039	0.9076
106	k__Bacteria;p__Planctomycetes;c__Phycisphaerae;o__Phycisphaerales;f__;g__	1	70	252.5	0.0042	0.6142
107	k__Bacteria;p__Planctomycetes;c__Phycisphaerae;o__Phycisphaerales;f__Phycisphaeraceae;g__	2	91	8	0.0044	0.9729
108	k__Bacteria;p__Planctomycetes;c__Phycisphaerae;o__WD2101;f__;g__	1	37	379.5	0.0040	0.0632
109	k__Bacteria;p__Planctomycetes;c__Pla3;o__;f__;g__	2	78	0	0.0039	0.9506
110	k__Bacteria;p__Planctomycetes;c__Planctomycetia;o__Gemmatales;f__Gemmataceae;g__	2	78	0	0.0039	0.8734
111	k__Bacteria;p__Planctomycetes;c__Planctomycetia;o__Gemmatales;f__Gemmataceae;g__Gemmata	1	55	285	0.0044	0.1695
112	k__Bacteria;p__Planctomycetes;c__Planctomycetia;o__Gemmatales;f__Isosphaeraceae;g__	2	71	0	0.0037	0.8132
113	k__Bacteria;p__Planctomycetes;c__Planctomycetia;o__Pirellulales;f__Pirellulaceae;g__	1	56	223	0.0045	0.2533
114	k__Bacteria;p__Planctomycetes;c__Planctomycetia;o__Pirellulales;f__Pirellulaceae;g__A17	1	3	39	0.0029	0.0034
115	k__Bacteria;p__Planctomycetes;c__Planctomycetia;o__Pirellulales;f__Pirellulaceae;g__Pirellula	1	48	139.5	0.0042	0.2556

116	k__Bacteria;p__Planctomycetes;c__Planctomycetia;o__Planctomycetales;f__Planctomycetaceae;g__Planctomyces	1	64	237	0.0045	0.3173
117	k__Bacteria;p__Proteobacteria;c__;o__;f__;g__	1	37	19	0.0041	0.1475
118	k__Bacteria;p__Proteobacteria;c__Alphaproteobacteria;o__Caulobacterales;f__Caulobacteraceae;g__	0	9	411	0.0023	0.0000
119	k__Bacteria;p__Proteobacteria;c__Alphaproteobacteria;o__Ellin329;f__;g__	1	45	95.25	0.0040	0.2660
120	k__Bacteria;p__Proteobacteria;c__Alphaproteobacteria;o__Rhizobiales;f__;g__	1	43	215	0.0039	0.0825
121	k__Bacteria;p__Proteobacteria;c__Alphaproteobacteria;o__Rhizobiales;f__Hyphomicrobiaceae;g__	1	49	151	0.0041	0.2011
122	k__Bacteria;p__Proteobacteria;c__Alphaproteobacteria;o__Rhizobiales;f__Hyphomicrobiaceae;g__Devosia	1	4	8	0.0028	0.0011
123	k__Bacteria;p__Proteobacteria;c__Alphaproteobacteria;o__Rhizobiales;f__Hyphomicrobiaceae;g__Rhodoplanes	1	47	57	0.0042	0.1117
124	k__Bacteria;p__Proteobacteria;c__Alphaproteobacteria;o__Rhodospirillales;f__;g__	1	18	176	0.0035	0.0158
125	k__Bacteria;p__Proteobacteria;c__Alphaproteobacteria;o__Rhodospirillales;f__Rhodospirillaceae;g__	1	106	119	0.0047	0.9317
126	k__Bacteria;p__Proteobacteria;c__Alphaproteobacteria;o__Sphingomonadales;f__Sphingomonadaceae;g__Sphingomonas	0	9	41	0.0019	0.0000
127	k__Bacteria;p__Proteobacteria;c__Betaproteobacteria;o__;f__;g__	2	87	1	0.0041	0.9522
128	k__Bacteria;p__Proteobacteria;c__Betaproteobacteria;o__Burkholderiales;f__Burkholderiaceae;g__Burkholderia	0	9	5	0.0019	0.0000
129	k__Bacteria;p__Proteobacteria;c__Betaproteobacteria;o__Burkholderiales;f__Comamonadaceae;Other	0	2	168	0.0012	0.0000
130	k__Bacteria;p__Proteobacteria;c__Betaproteobacteria;o__Burkholderiales;f__Comamonadaceae;g__Hydrogenophaga	0	9	790	0.0019	0.0000
131	k__Bacteria;p__Proteobacteria;c__Betaproteobacteria;o__Burkholderiales;f__Comamonadaceae;g__Limnohabitans	0	1	0	0.0010	0.0000

132	k__Bacteria;p__Proteobacteria;c__Betaproteobacteria;o__Burkholderiales;f__Comamonadaceae;g__Methylibium	0	2	334	0.0013	0.0000
133	k__Bacteria;p__Proteobacteria;c__Betaproteobacteria;o__Burkholderiales;f__Comamonadaceae;g__Polaromonas	0	2	498	0.0016	0.0000
134	k__Bacteria;p__Proteobacteria;c__Betaproteobacteria;o__Burkholderiales;f__Comamonadaceae;g__Variovorax	2	2	0	0.0028	0.0167
135	k__Bacteria;p__Proteobacteria;c__Betaproteobacteria;o__Burkholderiales;f__Oxalobacteraceae;g__	0	4	0	0.0016	0.0000
136	k__Bacteria;p__Proteobacteria;c__Betaproteobacteria;o__Burkholderiales;f__Oxalobacteraceae;g__Janthinobacterium	0	9	175	0.0019	0.0000
137	k__Bacteria;p__Proteobacteria;c__Betaproteobacteria;o__Burkholderiales;f__Oxalobacteraceae;g__Oxalobacter	0	4	0	0.0016	0.0000
138	k__Bacteria;p__Proteobacteria;c__Betaproteobacteria;o__Ellin6067;f__;g__	1	42	67	0.0040	0.0911
139	k__Bacteria;p__Proteobacteria;c__Betaproteobacteria;o__Gallionellales;f__Gallionellaceae;g__Gallionella	0	2	0	0.0023	0.0000
140	k__Bacteria;p__Proteobacteria;c__Betaproteobacteria;o__IS-44;f__;g__	2	76	129.75	0.0039	0.7997
141	k__Bacteria;p__Proteobacteria;c__Betaproteobacteria;o__MND1;f__;g__	1	87	273.75	0.0044	0.6779
142	k__Bacteria;p__Proteobacteria;c__Betaproteobacteria;o__Rhodocyclales;f__Rhodocyclaceae;g__Sulfuritalea	0	4	650	0.0028	0.0002
143	k__Bacteria;p__Proteobacteria;c__Betaproteobacteria;o__SBla14;f__;g__	0	6	2217	0.0030	0.0006
144	k__Bacteria;p__Proteobacteria;c__Deltaproteobacteria;o__;f__;g__	2	85	10	0.0040	0.9661
145	k__Bacteria;p__Proteobacteria;c__Deltaproteobacteria;o__Desulfobacterales;f__Desulfobulbaceae;g__	0	1	0	0.0023	0.0000
146	k__Bacteria;p__Proteobacteria;c__Deltaproteobacteria;o__Myxococcales;f__;g__	1	68	151	0.0043	0.4445
147	k__Bacteria;p__Proteobacteria;c__Deltaproteobacteria;o__Myxococcales;f__Haliangiaceae;g__	1	48	193.25	0.0042	0.2387
148	k__Bacteria;p__Proteobacteria;c__Deltaproteobacteria;o__NB1-j;f__;g__	2	90	53	0.0045	0.9128
149	k__Bacteria;p__Proteobacteria;c__Deltaproteobacteria;o__NB1-j;f__NB1-i;g__	2	91	23	0.0041	0.9854

150	k__Bacteria;p__Proteobacteria;c__Deltaproteobacteria;o__Syntrophobacterales;f__Syntrophobacteraceae;g__	2	90	0	0.0041	0.9954
151	k__Bacteria;p__Proteobacteria;c__Deltaproteobacteria;o__[Entotheonellales];f__[Entotheonellaceae];g__	2	65	61	0.0038	0.6642
152	k__Bacteria;p__Proteobacteria;c__Gammaproteobacteria;o__Alteromonadales;f__Alteromonadaceae;g__Cellvibrio	0	2	0	0.0016	0.0000
153	k__Bacteria;p__Proteobacteria;c__Gammaproteobacteria;o__Chromatiales;f__Ectothiorhodospiraceae;g__	1	1	0	0.0027	0.0024
154	k__Bacteria;p__Proteobacteria;c__Gammaproteobacteria;o__Pseudomonadales;f__Pseudomonadaceae;g__Pseudomonas	0	8	160	0.0020	0.0000
155	k__Bacteria;p__Proteobacteria;c__Gammaproteobacteria;o__Thiotrichales;f__Piscirickettsiaceae;g__	1	56	104.5	0.0043	0.2301
156	k__Bacteria;p__Proteobacteria;c__Gammaproteobacteria;o__Xanthomonadales;f__Sinobacteraceae;g__	1	96	130.5	0.0046	0.8135
157	k__Bacteria;p__Proteobacteria;c__Gammaproteobacteria;o__Xanthomonadales;f__Sinobacteraceae;g__Steroidobacter	1	33	27	0.0040	0.0559
158	k__Bacteria;p__Proteobacteria;c__Gammaproteobacteria;o__Xanthomonadales;f__Xanthomonadaceae;g__	1	28	188	0.0037	0.0303
159	k__Bacteria;p__Proteobacteria;c__Gammaproteobacteria;o__Xanthomonadales;f__Xanthomonadaceae;g__Luteimonas	1	6	0	0.0032	0.0046
160	k__Bacteria;p__Proteobacteria;c__Gammaproteobacteria;o__Xanthomonadales;f__Xanthomonadaceae;g__Lysobacter	2	54	89.25	0.0040	0.4860
161	k__Bacteria;p__Proteobacteria;c__Gammaproteobacteria;o__Xanthomonadales;f__Xanthomonadaceae;g__Thermomonas	1	33	647	0.0042	0.1270
162	k__Bacteria;p__SBR1093;c__;o__;f__;g__	2	75	20	0.0038	0.8882
163	k__Bacteria;p__Verrucomicrobia;c__Opitutae;o__Opitutales;f__Opitutaceae;g__	1	6	20	0.0032	0.0102

164	k__Bacteria;p__Verrucomicrobia;c__Opitutae;o__Opitutales;f__Opitutaceae;g__Opitutus	1	31	66	0.0037	0.0388
165	k__Bacteria;p__WS3;c__PRR-12;o__LD1-PA13;f__;g__	2	82	8	0.0040	0.9593
166	k__Bacteria;p__WS3;c__PRR-12;o__Sediment-1;f__;g__	2	84	2	0.0040	0.9811
167	k__Bacteria;p__WS3;c__PRR-12;o__Sediment-1;f__PRR-10;g__	2	77	3	0.0039	0.9126
168	k__Bacteria;p__WS3;c__PRR-12;o__wb1_H11;f__;g__	2	84	8	0.0040	0.9363
169	k__Bacteria;p__[Caldithrix];c__KSB1;o__Ucn15732;f__;g__	2	70	9	0.0038	0.7908

Supplementary Table 12 Information of 2 cm co-occurrence network of corrosion-related nodes

Id	Label	modularity_class	degree	betweenness	closeness	eigenvector
12	Acidobacteria-6__CCU21;g_unclassified	2	114	23.833	0.0043	0.8022
43	Solirubrobacterales;g_unclassified	0	105	44.25	0.0047	0.6395
46	Cytophagales;g_unclassified	1	8	3534	0.0031	0.0007
72	Chloroflexi;g_unclassified	0	127	2	0.0045	0.8856
89	Gemmatimonadetes;g_unclassified	0	115	117.25	0.0048	0.753
98	Nitrospira	2	87	45	0.0043	0.5259
145	Dechloromonas	1	10	8	0.0017	0
166	Xanthomonadales	1	10	1131	0.0025	4E-06
129	Magnetospirillum	1	7	5	0.0017	0
146	Rhodocyclaceae;g__Dok59	1	8	4	0.002	0
38	Nocardioidaceae;g_unclassified	1	5	304	0.0027	2E-05
119	Caulobacterales;g_unclassified	1	7	2	0.0017	0
123	Bradyrhizobium	1	11	359	0.0025	5E-06
125	Devosia	1	14	178	0.002	0
133	Hydrogenophaga	1	10	190.25	0.0017	0
134	Limnohabitans	1	12	324	0.002	0
135	Methylibium	1	6	4	0.0017	0
139	Gallionella	1	7	1182	0.0025	4E-06
147	Sulfuritalea	1	12	1	0.002	0
148	Gallionellales;g_unclassified	1	3	0	0.0016	0

169	Thermomonas	1	13	1	0.002	0
52	Sediminibacterium	1	1	0	0.002	0
137	Variovorax	1	3	0	0.002	0
140	o__IS-44;g_unclassified	2	60	414.5	0.0041	0.3557
143	Nitrosomonadales;g_unclassified	2	1	0	0.0028	0.0005
172	Opitutus	2	21	2969.5	0.0041	0.0837
