# Supplementary material for: Responses of soil microbiome to steel corrosion
Source: NPJ Biofilms Microbiomes. 2021 Jan 21;7:6. doi: 10.1038/s41522-020-00175-3 (PMC7820017; doi:10.1038/s41522-020-00175-3)
Supplement: Supplementary file 2 — Reporting Summary [file 41522_2020_175_MOESM2_ESM.pdf]

nature research

Corresponding author(s): Cheng-Ying Jiang, Shuang-Jiang Liu, Da-wei Zhang

Last updated by author(s): Nov 19, 2020

# Reporting Summary

Nature Research wishes to improve the reproducibility of the work that we publish. This form provides structure for consistency and transparency in reporting. For further information on Nature Research policies, see our Editorial Policies and the Editorial Policy Checklist.

## Statistics

For all statistical analyses, confirm that the following items are present in the figure legend, table legend, main text, or Methods section.

| n/a | Confirmed | |
|---|---|---|
| ☐ | ☒ | The exact sample size (*n*) for each experimental group/condition, given as a discrete number and unit of measurement |
| ☐ | ☒ | A statement on whether measurements were taken from distinct samples or whether the same sample was measured repeatedly |
| ☐ | ☒ | The statistical test(s) used AND whether they are one- or two-sided<br>*Only common tests should be described solely by name; describe more complex techniques in the Methods section.* |
| ☐ | ☒ | A description of all covariates tested |
| ☐ | ☒ | A description of any assumptions or corrections, such as tests of normality and adjustment for multiple comparisons |
| ☐ | ☒ | A full description of the statistical parameters including central tendency (e.g. means) or other basic estimates (e.g. regression coefficient) AND variation (e.g. standard deviation) or associated estimates of uncertainty (e.g. confidence intervals) |
| ☐ | ☒ | For null hypothesis testing, the test statistic (e.g. *F*, *t*, *r*) with confidence intervals, effect sizes, degrees of freedom and *P* value noted<br>*Give P values as exact values whenever suitable.* |
| ☒ | ☐ | For Bayesian analysis, information on the choice of priors and Markov chain Monte Carlo settings |
| ☒ | ☐ | For hierarchical and complex designs, identification of the appropriate level for tests and full reporting of outcomes |
| ☐ | ☒ | Estimates of effect sizes (e.g. Cohen's *d*, Pearson's *r*), indicating how they were calculated |

*Our web collection on statistics for biologists contains articles on many of the points above.*

## Software and code

Policy information about availability of computer code

| | |
|---|---|
| Data collection | Soil temperature, water content, and conductivity were measured in situ using a W. E. T. sensor (Eijkelkamp, Giesbeek, The Netherlands) |
| Data analysis | All statistical analyses were performed in R version 3.6.1. Code and detailed information on the computing steps are available on github (https://github.com/Rheannna/Responses-of-soil-microbiome-to-steel-corrosion) |

For manuscripts utilizing custom algorithms or software that are central to the research but not yet described in published literature, software must be made available to editors and reviewers. We strongly encourage code deposition in a community repository (e.g. GitHub). See the Nature Research guidelines for submitting code & software for further information.

## Data

Policy information about availability of data

All manuscripts must include a data availability statement. This statement should provide the following information, where applicable:

- Accession codes, unique identifiers, or web links for publicly available datasets
- A list of figures that have associated raw data
- A description of any restrictions on data availability

The datasets generated for this study can be found in NCBI with accession code PRJNA642359 (https://www.ncbi.nlm.nih.gov/bioproject/PRJNA642359)

# Field-specific reporting

Please select the one below that is the best fit for your research. If you are not sure, read the appropriate sections before making your selection.

☐ Life sciences ☐ Behavioural & social sciences ☒ Ecological, evolutionary & environmental sciences

For a reference copy of the document with all sections, see nature.com/documents/nr-reporting-summary-flat.pdf

# Ecological, evolutionary & environmental sciences study design

All studies must disclose on these points even when the disclosure is negative.

| | |
|---|---|
| Study description | To study the interaction between the soil microbiome and metals during the corrosion process, burial tests were conducted in natural soil environments using different engineering materials, including a widely used carbon steel variant (Q235), a copper-bearing pipeline steel (X80Cu), and a polyethylene (PE) control, which is commonly used in inert protective coatings for underground pipelines. |
| Research sample | The buried materials and the surrounding soil samples were retrieved. The surrounding soil microbial communities were studied using high-throughput sequencing. Comparative analysis of the microbial diversity and community assembly between soil samples collected at 2 cm and 10 cm horizontal distance from the materials was conducted. The major environmental determinants of ecological distribution of prokaryotic taxa were identified. The bioindicators of Q235 and X80Cu corrosion were recognized using random forest modeling, and the potential metabolic pathways associated with the corrosion process were determined through metagenomics prediction. Two co-occurrence microbial networks were constructed to elucidate the interaction between the corrosion-inducing microorganisms and to determine the possible mechanism underlying corrosion in the soil environment. |
| Sampling strategy | A 4 m × 4 m square plot was selected and excavated to an approximate horizontal distance of 2 m from the ground. The materials used in this experiment were cut into 5 cm × 8 cm × 1 cm coupons. All the metal coupons were abraded, polished, cleaned, and weighed. 19 sample points were set up at 50 cm intervals and marked as a1-a7, b1-b7, and c1-c5. The soil samples S1-S19 were collected at the corresponding positions of the sample points. Concurrently, the Q235 carbon steel coupons Q1-Q7 were placed vertically at positions of a1-a7 (7 individuals), X80Cu pipeline steel coupons X1-X7 at b1-b7 (7 individuals), and polyethylene polymer materials at c1-c5 (5 individuals). After the burial operation was completed, the upper layer of soil was buried back into the plot. After 5 months of experiment burial, the corresponding positions of the Q1-Q4 and X1-X4 samples were excavated. The metals were stored in place and samples were collected from soil at a horizontal distance of 2 cm from Q235 (named Qs2_1-Qs2_4) and X80Cu (named Xs2_1 -Xs2_4) surfaces, and at a horizontal distance of 10 cm from Q235 (named Qs10_1-Qs10_4) and X80Cu (named Xs10_1-Xs10_4) surfaces. Concurrently, temperature, moisture, and electrical conductivity values at each sampling point were measured immediately. Next, the buried materials were removed. After 10 months of experimental burial, the corresponding positions of Q5-Q7, X5-X7, and P1-P5 were excavated. The sampling method was the same as that mentioned above. Samples were collected from soil at a horizontal distance of 2 cm from Q235 (named Qs2_5-Qs2_7), X80Cu (named Xs2_5-Xs2_7), and polyethylene (named Ps2_1-Ps2_5) surfaces; samples were also collected at a horizontal distance of 10 cm from Q235 (named Qs10_5-Qs10_7), X80Cu (named Xs10_5-Xs10_7), and polyethylene (named Ps10_1-Ps10_5) surfaces. Eventually, 56 soil samples and 15 metal samples were obtained. The test metals were weighed and the redox potential values were measured. Soil samples were collected in sterile plastic bags using a shovel and were transported to the laboratory within 4 h. DNA extractions for microbial analysis were completed within two days and the soil samples for physical and chemical characterization were stored at 4 °C until used. |
| Data collection | Soil temperature, water content, and conductivity were measured in situ using a W. E. T. sensor (Eijkelkamp, Giesbeek, The Netherlands) |
| Timing and spatial scale | The Yangfang Corrosion Test Station (116°16' E, 39°59' N), Changping District, Beijing was selected for conducting the metal material corrosion experiments under natural conditions. The soil samples S1-S19 were collected at the corresponding positions of the sample points at 06, 2016. Soil samples named Qs2_1-Qs2_4, Xs2_1 -Xs2_4, Qs10_1-Qs10_4 and Xs10_1-Xs10_4 and buried materials named Q1-4 and X1-4 were collected at 11, 2016. Soil samples named Qs2_5-Qs2_7, Xs2_5 -Xs2_7, Qs10_5-Qs10_7 and Xs10_5-Xs10_7 and buried materials named Q5-7 and X5-7 were collected at 04, 2017. |
| Data exclusions | No data were excluded |
| Reproducibility | Seven copies of X80Cu and Q235 and five copies of PE were used for biological reproduction |
| Randomization | Group of individuals was done according to sampling time and material type. |
| Blinding | not relevant: there was no treatment of any living animals/humans in our experiment |

Did the study involve field work? ☒ Yes ☐ No

## Field work, collection and transport

| | |
|---|---|
| Field conditions | The Yangfang Corrosion Test Station had a typical warm temperate, sub-humid, continental monsoon climate. The average annual temperature was 11.8 °C and the average precipitation was 550–600 mm, concentrated in summer. Temperature was 33.9, 19.66 and 11.5 °C for three sampling time separately |

| | |
|---|---|
| Location | The Yangfang Corrosion Test Station (116°16' E, 39°59' N), Changping District, Beijing was selected for conducting the metal material corrosion experiments under natural conditions. |
| Access & import/export | Permissions for scientific research and sampling at that site including the burial operation of sample material had been granted by National Environmental Corrosion Platform |
| Disturbance | After sampling collection was completed, the upper layer of soil was buried back into the plot |

# Reporting for specific materials, systems and methods

We require information from authors about some types of materials, experimental systems and methods used in many studies. Here, indicate whether each material, system or method listed is relevant to your study. If you are not sure if a list item applies to your research, read the appropriate section before selecting a response.

## Materials & experimental systems

| n/a | Involved in the study | |
|---|---|---|
| ☒ | ☐ | Antibodies |
| ☒ | ☐ | Eukaryotic cell lines |
| ☒ | ☐ | Palaeontology and archaeology |
| ☒ | ☐ | Animals and other organisms |
| ☒ | ☐ | Human research participants |
| ☒ | ☐ | Clinical data |
| ☒ | ☐ | Dual use research of concern |

## Methods

| n/a | Involved in the study | |
|---|---|---|
| ☒ | ☐ | ChIP-seq |
| ☒ | ☐ | Flow cytometry |
| ☒ | ☐ | MRI-based neuroimaging |
